# Transposable Elements: Distribution, Polymorphism, and Climate Adaptation in *Populus*

**DOI:** 10.3389/fpls.2022.814718

**Published:** 2022-02-01

**Authors:** Yiyang Zhao, Xian Li, Jianbo Xie, Weijie Xu, Sisi Chen, Xiang Zhang, Sijia Liu, Jiadong Wu, Yousry A. El-Kassaby, Deqiang Zhang

**Affiliations:** ^1^National Engineering Laboratory for Tree Breeding, College of Biological Sciences and Technology, Beijing Forestry University, Beijing, China; ^2^Key Laboratory of Genetics and Breeding in Forest Trees and Ornamental Plants, Ministry of Education, College of Biological Sciences and Technology, Beijing Forestry University, Beijing, China; ^3^Department of Forest and Conservation Sciences, Forest Sciences Centre, Faculty of Forestry, The University of British Columbia, Vancouver, BC, Canada

**Keywords:** transposable elements, Helitron transposons, 24 nt siRNA, forest genetic resources conservation, adaptive evolution

## Abstract

Transposable elements (TEs) are a class of mobile genetic elements that make effects on shaping rapid phenotypic traits of adaptive significance. TE insertions are usually related to transcription changes of nearby genes, and thus may be subjected to purifying selection. Based on the available genome resources of *Populus*, we found that the composition of Helitron DNA family were highly variable and could directly influence the transcription of nearby gene expression, which are involving in stress-responsive, programmed cell death, and apoptosis pathway. Next, we indicated TEs are highly enriched in *Populus trichocarpa* compared with three other congeneric poplar species, especially located at untranslated regions (3′UTRs and 5′UTRs) and Helitron transposons, particularly 24-nt siRNA-targeted, are significantly associated with reduced gene expression. Additionally, we scanned a representative resequenced *Populus tomentosa* population, and identified 9,680 polymorphic TEs loci. More importantly, we identified a Helitron transposon located at the 3′UTR, which could reduce *WRKY18* expression level. Our results highlight the importance of TE insertion events, which could regulate gene expression and drive adaptive phenotypic variation in *Populus*.

## Introduction

Transposable elements (TEs), which are known as a source of genetic variation (McClintock, 1984), and its composition is extremely different in diverse species (Le Rouzic et al., 2007; Barron et al., 2014; Le et al., 2015; Li et al., 2018). TEs are mobile genetic sequences, with known two major categories: Class I retrotransposons and Class II DNA transposons (Wicker et al., 2007). Intriguingly, different transposons often form the major branches in diverse species. For example, there were less than a dozen long terminal repeat (LTR) retro-families in barley (Thiel et al., 2009), and the outbreak of retrotransposon activity shaped the current state of its reference genome in rice (El Baidouri and Panaud, 2013). Abundant information about transposon activity can be obtained by comparing closely related species (Brookfield, 2005; Agren et al., 2014; Barron et al., 2014), which will contribute to an in-depth understanding of the frequency and impact of TE activities in biological evolution. TEs play an important role in the formation of genomic structure and phenotypic variation in different organisms (Feschotte et al., 2002; Ellinghaus et al., 2008; Feschotte, 2008). Moreover, sequence and genomic distribution variability produced by TEs had become sources for regulatory elements (Feschotte, 2008). Therefore, the TE insertions can regulate the gene expression level through *cis-* or *trans*- regulatory elements located in TE sequences, or through epigenetic modification caused by TE insertions or deletions (Chung et al., 2007; Gonzalez et al., 2009; Naito et al., 2009; Hollister and Gaut, 2011; Bousios et al., 2016; Stuart et al., 2016). In addition, TEs carry their own promoters and induce the expression of nearby genes by reading or transcription (Feschotte, 2008; Naito et al., 2009; Pereira et al., 2009). In maize, a transposon located 58.7–69.5-kb upstream of domesticated gene *teosinte branched1* (*tb1*) was an enhancer of gene expression, which explained the reason why the dominance of maize root tip was higher than its progenitor gene (Studer et al., 2011). In oil palm, the methylation deletion of *karma* transposon in the *DEFICIENS* intron leads to the origin of covered somatic clonal variation (Kok et al., 2015; Ong-Abdullah et al., 2015).

Specially, TEs may serve as a medium for rapid adaptation, because they can quickly create genetic diversity (Oliver et al., 2013; Schrader et al., 2014). Although the evolutionary forces controlling the accumulation or removal of TE between generations are not completely clear, it is known that the activity of TE in plants is inhibited by epigenetic pathways (Slotkin and Martienssen, 2007; Bousios et al., 2016; Stuart et al., 2016). These pathways require small interfering RNA (siRNAs) to target specific TE insertion through sequence consistency (Almeida and Allshire, 2005; Hollister and Gaut, 2011; Felippes et al., 2012). siRNAs with a length of 20–30 nucleotides are cleaved by different DCL proteins from dsRNAs, and finally defined by 21–24 nucleotides size (Brant and Budak, 2018). The 24-nt siRNAs target DNA methylation such as *met1* and *ddm1* mutants that decreased TE methylation levels, with concomitantly increased the expression and activity of some TEs (Lippman et al., 2004; Jia et al., 2009; Tsukahara et al., 2009). The first discovery of TE is due to their impact on phenotype and the extent to which they are ubiquitous in the genome. Until the emergence of whole genome sequencing, our vision has expanded from the selection of several transposons to the whole genome of many species that can be used to release genome sequences. Whole genome sequencing reveals the involvement of TE at the genomic structure level, and these results provide insights into the functional degree of these TE sequences. Although TEs play an important role in the formation of genomic structure and phenotypic variation, TEs in a positive selection state in natural plant populations are largely unknown. Forests cover most of the world’s land surface and play a vital role in the evolution of biodiversity and function of forest ecosystem (Su et al., 2018). Poplar is one of the main woody plant model systems. Therefore, it is very important to study which genes are regulated by TE insertions to promote its rapid adaptation to climate change (Pecinka et al., 2010; Cavrak et al., 2014; Pietzenuk et al., 2016). The natural phenotypic variation of *Populus tomentosa* is distributed in the vast geographical area of northern China (30°N–40°N, 105°E–125°E), indicating poplar adaptive evolution (Du et al., 2018). In recent studies, DNA sequencing technology promotes the assessment of genetic diversity and provides a useful basis for natural populations, indicating forest genetic resources conservation is imperative (Su et al., 2018). Here we investigated TE abundance, 24-nt-siRNA targeting, and their important roles on gene expression level in *Populus trichocarpa*. Then, we identified polymorphic TEs from a representative *P. tomentosa* population, and illustrated TEs differential expansion in different climatic regions. We identified one Helitron type TE insertion which is a likely candidate to playing a role in adaptive evolution, and its insertion in 3′UTR could repress gene expression. Overall, our study highlights the potential impact of TEs on the adaptive evolution of natural population in poplar.

## Materials and Methods

More detailed information on the materials and methods used in this study were provided in Supplementary Method 1.

### Identification and Annotation of Polymorphic Transposable Element Sites

The *de novo* annotation of TEs using RepeatModeler v2.0.1 with the parameters “ncbi” and “LTRStruct” to generate TE family consensus sequences. We then excluded unknown TE consensus sequences by TEclass v2.1.3 with default parameters (Abrusan et al., 2009). Remaining consensus sequences were assigned to defined TE superfamilies for next steps. The identified repeats in *Populus trichocarpa* (Tuskan et al., 2006), *Populus tomentosa* (CRA000903), *Populus alba* × *Populus glandulosa* (84K; Qiu et al., 2019), and *Populus alba* (Liu et al., 2019) genomes were appended to RepBase (RM Database; Version: 2017-01-27) and Tandem Repeat Finder (TRF; v4.09), resulting to be annotated by RepeatMasker v4.0.6^1^ with using ncbi blast version 2.10.0+ as blast engine (Camacho et al., 2009). To reduce false positives, we retained alignments of sequence identity over 80%, as described previously (Hollister and Gaut, 2011; Niu et al., 2019). Polymorphic TEs were identified using TEPID (Stuart et al., 2016) based on the raw reads of 435 resequencing genomes of *P. tomentosa*, resulting to be divided into three subpopulations.

### Clustering Analysis of *Populus tomentosa* Accessions

In 2011, 435 unrelated individuals of *P. tomentosa* were asexually propagated in Guan County, Shandong Province, China (36°230N, 115°470E) (Du et al., 2018), representing almost the entire species natural distribution (30–40°N, 105–125°E). These individuals are divided into distribution areas in the South (S), Northwest (NW), and Northeast (NE; Huang, 1992). Due to the randomness and repeatability of data, we used Python script to randomly draw individuals among different climatic regions. Finally, raw paired-end reads of 87 accessions nearly representing *P. tomentosa* entire natural range were used in this study, including 23, 35, and 29 from NE, NW, and S, respectively (Supplementary Table 2). The raw reads were processed to clean reads and then mapped to the *P. tomentosa* reference genome using BWA (version: 0.1.17) with default parameters (Li and Durbin, 2009). Single nucleotide polymorphisms (SNPs) were invoked using the Genome Analysis Toolkit (GATK version: 4.0) (DePristo et al., 2011) with a mass value of 25 as the threshold. Then use EIGENSOFT (version: 7.2.1) for principal component analysis (PCA) with minor allele frequency (MAF) ≥ 0.05 (Price et al., 2006).

### Detection of Adaptive Transposable Element Insertions

The average number of pairwise differences at each locus between any two sequences, π (Nei, 1987), was used for nucleotide diversity calculations. In addition, after screening the deletion values, Tajima’s D of the neutral test was estimated using genotype data (Tajima, 1989). Based on genetic polymorphism data, population differentiation Statistics (*F*_*st*_) were performed for each paired region differentiation with a 5,000-bp step size (Danecek et al., 2011). More details on the statistics of *F*_*st*_, π, and Tajima’s D calculations are provided in Supplementary Method 1, and 5,000-bp were always used as step-length. We used the first 5% of the empirical distribution of *F*_*st*_ and π values in the polymorphic region as candidates to represent the characteristics of significant difference selection between each polymorphic region in the subgroup (popNE vs. popNW, popNE vs. popS, and popNW vs. popS). Selection sweeps were evaluated using a 500-bp non-overlapping windows for reduction of diversity (ROD) statistical adjustment method, and an extensive 10-kb flanking region of each polymorphic TE region (Xu et al., 2011). A significant low π value (low ratio value) is positive selection index of TE insertion allele which were considered as putatively candidates. Then, Tajima’s D was estimated in 20-kb flanking regions for the candidate adaptive TEs. All polymorphic TEs in the selective sweep regions were identified as adaptive TE insertion under selection. Details were described in Supplementary Method 1.

### Identification of Transcription Factor Binding Sites and Enriched Motifs

Transcription factor binding sites (TFBS) were predicted by PlantPAN 2.0 (Chow et al., 2016) and PlantTFDB 4.0 (Jin et al., 2017). AME integrated in the MEME suite was used for motif enrichment analysis (Bailey et al., 2015). For enrichment analysis, Genomic regions were randomly selected as the control background for enrichment analysis, and only motifs with *P* < 0.05 (Fisher exact test) were regarded as significant. Each *cis*-element was evaluated for positional bias using two common approaches: the clustering factor (*CF-score*) (FitzGerald et al., 2004), and a recently proposed index, the *Z-score* (FitzGerald et al., 2004; Ma et al., 2013).

### Identification of 24*-*nt siRNA Loci

The 24-nt siRNA loci were re-mapped and compared using Pln24NT database and method described by Liu et al. (2017). Non-redundant 24-nt siRNA sequences using Bowtie v1.2 (-v 1 -a -m 50 –best –strata) mapping to the reference genome (Langmead, 2010). We used ShortStack v3.8.4 to select siRNA clusters with a minimum coverage of 10 reads (Axtell, 2013). For 24-nt siRNA clusters, if they exist in a 150-bp window, they are merged to generate the final set of 24-nt siRNA loci (El Baidouri et al., 2015). Furthermore, we mapped siRNA loci to genomic locations and labeled at least one 24-nt siRNA matched single TE as siRNA+; TEs without matching siRNA loci were labeled as siRNA−.

### Gene Expression of *Populus trichocarpa* Under Heat Stress

Total RNAs were extracted from 1-year-old *P. trichocarpa* at 0, 4, 8, 12, 24, 36, and 48 h after exposure to 40°C heat treatment with three technical replicates per sample. The method for estimating the genes expression levels by fragments per kilobase of exon per million (FPKM) reads mapped is described in Trapnell et al. (2012). In total, the expression levels of 17,003 genes were measured across different treatment time points. The expressed datasets used in this study were published by our previous study with accession number CRA001776 available at the BIGD Genome Sequence Archive^2^.

### Real-Time Reverse Transcription Polymerase Chain Reaction Analyses

When cDNA was synthesized using superscript II reverse transcriptase (Invitrogen), about 2.0 mg of total RNA was used. 1.0 ml of cDNA, diluted twice fold, and analyzed with SYBR premix ex Taq (Takara). Delta-Delta CT quantitative method was used to assess the difference between repetitions. The ACTIN gene (*Actin1*, Accession number: EF145577) and 18S were used as internal controls. PCR conditions included an initial denaturation step at 95°C for 3 min, then 40 cycles at 95°C for 30 s, 1 min at 60°C, 30 s at 72°C, and finally an extension of 5 min at 72°C. Primers used for Real-time reverse transcription polymerase chain reaction (RT-qPCR) in our study are listed in Supplementary Table 7.

### Transient Luciferase Activity Assays

We amplified full-length *WRKY18* DNA, full-length *WRKY18* without the 3′UTR, and the full-length *WRKY18* without the Helitron from *P. tomentosa* clone “1316” using the primers listed in Supplementary Table 7. All amplified fragments were cloned into pCAMBIA1301H, where the *WRKY18* genes were driven by its promoter region (2-kb fragments of upstream of the translational start site). The *WRKY18* 3′UTR deletion mutants (Del1, Del2) were generated from the full-length *WRKY18* 3′UTR with the primers. Details were described in Supplementary Method 1. *Arabidopsis* protoplast preparation and transient expression assay were performed as previously described (Yoo et al., 2007). The luciferase (LUC) assay and the method of calculating the relative expression levels were detailed describe in Supplementary Method 1.

## Results

### Distribution of Transposable Elements Among Four Poplar Whole Genomes

To study the genetic variation and evolutionary relationship of TEs in *Populus*, we investigated four poplar species, resulting in 162,517∼212,595 TE insertions (average covering a total of 167.98 Mb; Supplementary Table 1). Additionally, transposons were observed to be enriched in the centromere region, and the distribution of TEs and genes were negatively correlated on chromosome arms across the four studied species (Supplementary Figures 1A,B). No significant differences of TE and gene density were detected between these species, with a median TE density of 22-kb per 100-kb window across the chromosome [Mann–Whitney *U* test (MWU), *P* = 0.064], and gene density averaged 0.33 (MWU, *P* = 0.087). Meanwhile, the median TE density of *P. trichocarpa* chromosome arms was 24-kb per 100-kb window (0.24), illustrating that more *P. trichocarpa* genes are closer to TEs than that of the other three genomes (MWU, *P* < 0.01; Supplementary Figure 2E). Notably, we detect more genes harboring TE insertions in *P. trichocarpa* than the other three genomes (11.06%; 8.48–10.81%), demonstrating that TE insertions might contribute to the diversification of gene expression in *Populus*. Detailed analyses revealed that 3.92% (or 1,392) of *P. trichocarpa* genes have a TE insertion located within 5′ and 3′ of the coding region, with a larger proportion than the other species (0.45–0.62%) (Supplementary Figures 2A,B; Fisher’s exact test; *P* < 0.01), suggesting that TEs insertions had a higher potential of affecting genes in *P. trichocarpa.* In addition, three super-families of elements, Gypsy, Copia and Helitron, showed a particularly striking accumulation in the genic regions of *P. trichocarpa* (Supplementary Figures 2A–D). Especially, Helitron elements had higher frequency in introns and exons (Supplementary Figure 2C), suggesting that they may be under strong purifying selection.

**FIGURE 1:**
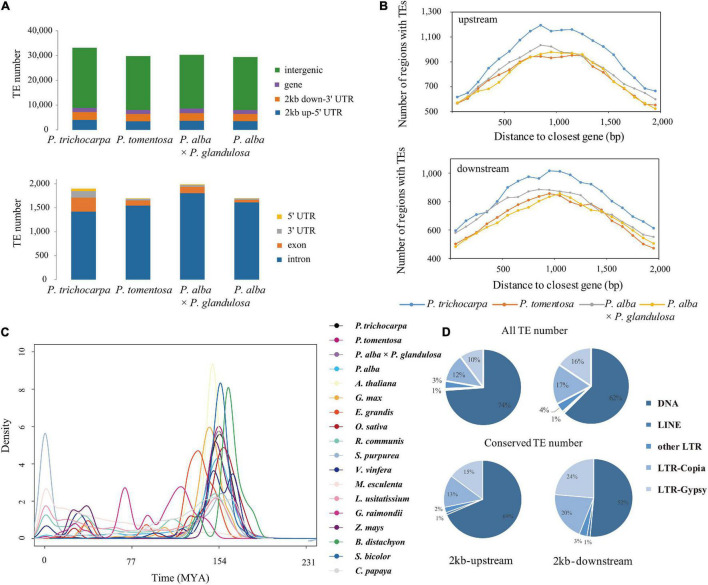
Distribution of transposable elements in *Populus*. **(A)** Number of TEs in different genic regions of *Populus trichocarpa, P. tomentosa, P. alba* × *P. glandulosa* (84K), and *P. alba* genomes. **(B)** TEs enriched in 100-bp bins in the 2-kb upstream and 2-kb downstream region of genes between collinear gene pairs of *P. trichocarpa, P. tomentosa*, 84K, and *P. alba*. The *x*-axis represents the distance from TEs to the start codon (stop codon) of the nearest gene; the *y*-axis represents the number of TEs genes at different distances from the start codon (stop codon). **(C)** Density distributions of the Ks values for LTR transposons of 18 different species. **(D)** Distribution of transposable elements in *P. trichocarpa* of all TEs and conserved TEs (collinear TEs among four poplar genomes) in 2-kb upstream and 2-kb downstream regions.

To study the genetic effect of genomic TEs, we first identified 25,321 conserved TEs among the four studied species (Supplementary Data 2). Among them, 84.03–95.72% (22,218 on average) TEs were highly enriched in intergenic regions (Supplementary Data 3–6), of which 29.43–31.09% were located within 2-kb genes flanking regions. Notably, 1,283 (18.76–19.75%) were classified as Gypsy super-family, 1,109 (16.28–16.99%) were classified Copia super-family, and a large number of DNA transposons, especially Helitron elements (3,014; 44.46–45.87%; Figure 1 and Supplementary Data 3–6). Of these DNA transposons, 13.89–15.70% were located within promoters and 11.40–12.61% were in 2-kb-downstream of genes (Figures 1A,B). Additionally, we found that Ty1/Copia (26.27–27.78%) and Gypsy (26.03–26.79%) families were frequently located within genes, compared to other TE families (Supplementary Figures 2, 3), indicating that LTR might potentially contribute to the diversification of gene expression in *Populus*. Moreover, an explosive of evolution time indicated by LTR of 18 different species revealed evidence for any species- or section-specific family members (Figure 1C), demonstrating that the LTR complement correlated with the divergence of these species. Furthermore, gene ontology enrichment analysis of collinear genes affected by TEs suggested that these genes were involved in stress-responsive (GO:0016301), programmed cell death (GO:0012501), apoptosis (GO:0006915), cell death (GO:0008219), and death (GO:0016265) were particularly affected by these TEs (Supplementary Figure 4). These findings suggested that TEs, the lineage-indistinctive components of genomes, might drive the diversification of gene regulation.

### Transposable Elements and Small RNAs Contribute to the Divergence of Gene Expression

**FIGURE 2:**
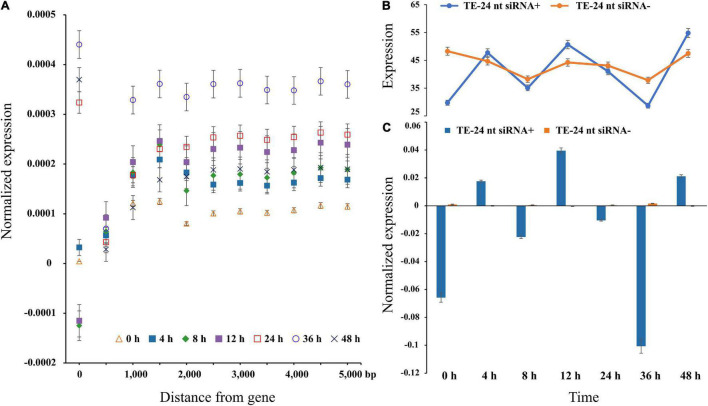
Gene expression level in each bin farther away from the nearest TE in *P. trichocarpa.* **(A)** The average value of gene expression was TE distance, with 500-bp bin and max to 5000-bp. A distance of zero indicates TEs in intron or UTRs. **(B,C)** Gene expression levels of TEs (distances binned in 1000-bp) with and without matching 24-nt siRNAs.

To explore TEs regulation on nearby genes expression, we used the publicly available RNA-seq data. Therefore, the average gene expression level increased with the distance from the nearest TE, and the gene expression level reached the maximum when the nearest TE distance is 1-kb (Figure 2A). Under high temperature treatment, the difference dissipated within a distance of ∼1-kb from the gene across different treatment time points, which confirmed that TEs were more likely to be active under stress treatments. Additionally, the expression at 0 h is significantly lower than 36 h (Student’s *t-*test; *P* < 0.01), indicating that temperature could affect transposon activity (Figure 2). Remarkably, the expression levels of genes with TEs were uniformity ablated and began to stabilize at 1-kb (Figure 2).

One of the many factors affecting TEs accumulation near genes is siRNA guided transcriptional gene silencing. To test this possibility, 7,868,825 24-nt siRNA sequences were clustered to 56,840 siRNA loci, of which 44,735 loci (∼78.70%) perfectly matched the TE sequence. We observed that the expression level was only fluctuated significantly for TEs-24-nt siRNA+ (Student’s *t*-test; *P* < 0.01), and not for TEs-24-nt siRNA− (Student’s *t*-test; *P* = 0.3). At 0 h, the average expression levels of genes with flanking TEs-24-nt siRNA+ were twofold higher than TEs-24-nt siRNA− (*P* < 0.05). The overall pattern is clear: proximal TEs are related to gene expression level. When TEs are targeted by 24-nt siRNAs, the decline or increasing of expression is statistically supported.

### Regulatory Motifs Highly Enriched in Conserved Transposons

To investigate the TE insertions putative regulatory mechanisms, we investigated 25,681 TEs insertions with 246 enriched motifs which were found in promoters of protein encoding genes among the four studied poplar species (Supplementary Data 8). The typical CAAT box and TATA box are very important for transcription initiation and occur in all promoter regions. In addition, six motifs were found to be enriched within 2-kb of the transcription start sites (TSS; *P* < 0.01; Supplementary Figure 5), revealing that decaying TE sequence might provide *cis* regulatory elements. Top-scoring *cis*-elements in TE insertion regions, such as the WRKYGQK motif (CGTTGACTWWDDYWDWNHH), CACG sequences (AAAGTCAACGN), ethylene-responsive elements (YREGIONNTPRB1B), and W-box (WBOXATNPR1), all having a peak position within 200-bp upstream of TSS, a high *Z-score* (>3) and/or *CF* score, or are highly overrepresented compared with random sequences reaffirming some extent of conservation in plant promoter architecture and TE insertions (Supplementary Figure 5 and Supplementary Data 8). Further analysis identified that the motifs were associated with functions of defense response (GO:0006952), and response to chitin (GO:0010200) (Supplementary Data 7).

**FIGURE 3:**
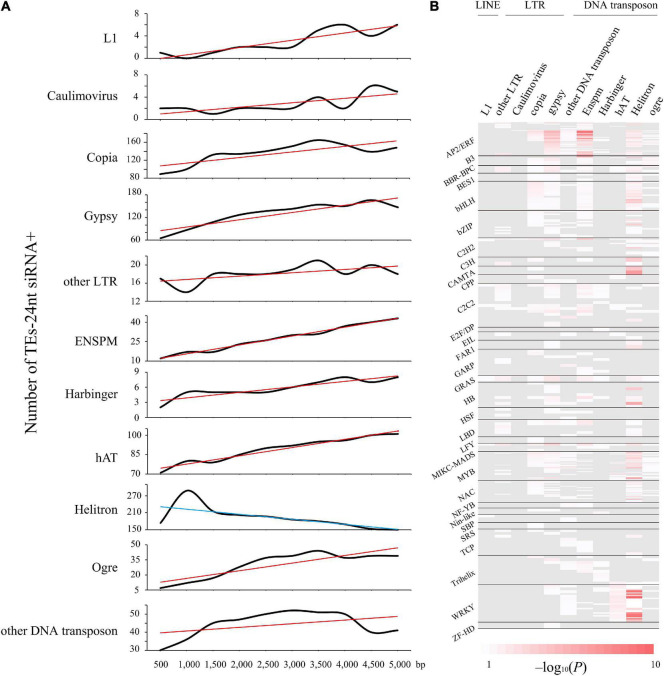
Helitron transposons drive 24-nt siRNAs as a supply of TFBS. **(A)** The trend of quantity variation of TEs with matching 24-nt siRNAs. TEs distance is in 500-bp bin, up to 5,000-bp. Red and blue lines represent the increasing and declining trend, respectively. **(B)** Distribution peaks of *cis*-regulatory elements enriched in the different types of TEs, showing the distribution profile of motifs from 31 transcription factor families significantly (*P* < 0.05) enriched in the Helitron type transposons.

Similarly, conserved TEs are more abundant in genomic neighborhoods of stress response and immune-related genes (Supplementary Figure 4). For instance, we uncovered a intronic Copia-containing the gene Potri.001G152100, which is involved in endoplasmic reticulum membrane organization that was considered as stress responses (Ascencio-Ibáñez et al., 2008). In addition, Helitron DNA transposons were more enriched with TFs than others, such as WRKY, MYB, and AP2/ERF families (Figure 3B and Supplementary Data 9). Likewise, 29.52% Helitron transposons insertions within LRR-NB-ARC related genes, which confer broad resistance to biotic and abiotic stresses, are associated with transcript truncation and could therefore cause increased susceptibility. Additionally, we measured the trend of different TE families abundance within 24-nt siRNAs from 0∼5-kb distance to nearby genes (Figure 3A). Intriguingly, only Helitron DNA transposons had a downward trend with the increasing distance and reached peak at 1-kb, indicating that Helitron transposons made the most significant contribution to donate *cis*-elements and regulated nearby genes with 24-nt siRNAs targeted (Figures 2, 3). Conversely, other TE families with 24-nt siRNAs targeted might affect gene regulation by TEs copy and movement. These findings indicated that the insertion of TEs, especially Helitron, affected the expression of adjacent genes by providing new regulatory signals.

### Polymorphic Transposable Elements Insertions Affect Natural Variation in Three Sub*-*Populations From Different Climatic Zones of *Populus tomentosa*

**FIGURE 4:**
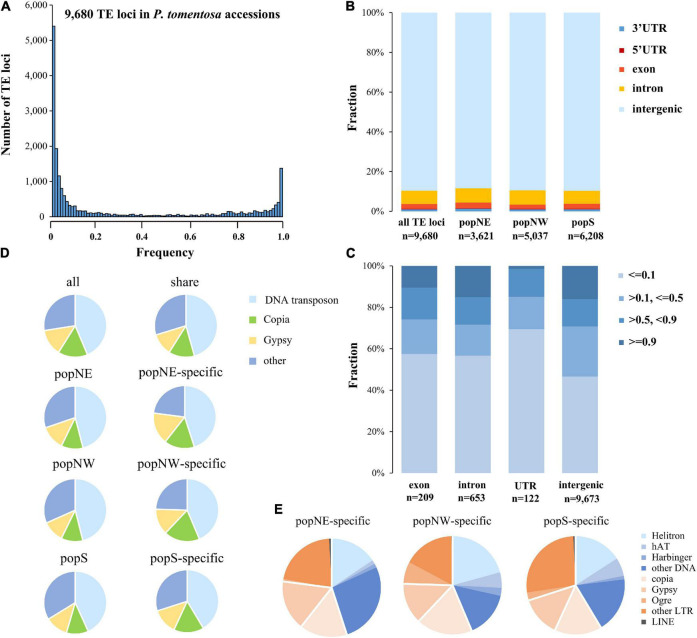
Frequency and distribution of polymorphisms TEs in *P. tomentosa* population. **(A)** Frequency distribution of polymorphic TEs in the genomes of *P. tomentosa* natural population. The *x*-axis represents the number of additions with the same TE insertion. **(B)** Distribution pattern of polymorphic TEs in the genome. **(C)** Frequency of polymorphic TEs located in different genomic regions. **(D)** The composition of polymorphic TEs in different groups (including all groups, shared groups, and specific groups). **(E)** Composition of different types of polymorphic TEs in different specific sub-populations.

Based on the inconsistency between mapping distance and read pair insertion size, we used short-read whole-genome resequencing data of *P. tomentosa* accessions representing three sub-populations, resulting in a total of 9,680 polymorphic TE loci (Supplementary Table 2, Supplementary Figures 6A, 7A, and Supplementary Data 10). Further, we validated 12 identified TE loci by PCR sequencing by using 12 accessions from three subpopulations (Supplementary Table 6), leading in a U-shaped of TEs frequency distribution which supported by a previous study (Robert et al., 2015). Significant correlations between genetic distance and polymorphic TE insertions number were detected in our study (Person correlation; *r* = −0.25, *P* > 0.01; Figure 4A), among these 5,370 loci were shared. Among all three subpopulations, 3,621, 5,037, and 6,208 polymorphic TE loci in popNE, popNW, and popS, respectively (Supplementary Data 11–13). Additionally, we identified 213, 685, and 1,401 population-specific TE loci in popNE, popNW, and popS, respectively (Supplementary Table 3), proving that popS had the highest number of polymorphic TEs, population-specific TEs and the highest number of inserted TEs (Supplementary Table 3 and Supplementary Data 13). Furthermore, popNE, popNW, and popS are generally consistent in the composition of polymorphic TE types and the polymorphic TE shared among them. Most of the polymorphic loci are distributed in the intraspecific or specific population level intergenic regions, but nearly 10% of the polymorphic loci still exist in the gene regions of coding sequences or introns (Figure 4B). Totally, 245 polymorphic TE insertions distributed in CDS region of 194 genes (Supplementary Data 14), which were enriched in defense response, response to stresses, RNA biosynthetic process, and immune response (GO enrichment analysis, FDR < 0.05). Compared with the TE located at intergenic regions, the polymorphic TEs inserted into CDS regions and untranslated regions (UTRs) were significantly biased to low frequency (frequency ≤ 0.1) (Figure 4C; Fisher’s exact test, multiple testing corrected *P* < 0.01 for TEs in CDS regions and UTR regions), indicating that the diffusion of TE insertions in CDS and UTR regions are limited by purification selection (Figure 4D and Supplementary Table 4). Of the 9,680 polymorphic TEs, the proportion of DNA transposon-type TEs (46.02%) is substantially higher than that of Gypsy-type TEs (11.39%) and Copia-type TEs (11.85%). Additionally, DNA transposon-type TEs (26.84%) were the most enriched in popNE-specific polymorphic TEs, whereas Helitron-type TEs (20.73%) and other LTR-type (26.55%) were the major components in popNW, and popS-specific polymorphic TEs, respectively (Figure 4E and Supplementary Table 4). Overall, the expansion and contraction of different TEs types differentiated among the three *P. tomentosa* sub-populations.

### Helitron Inserted at 3′UTR Repressed *WRKY18* Expression

**FIGURE 5:**
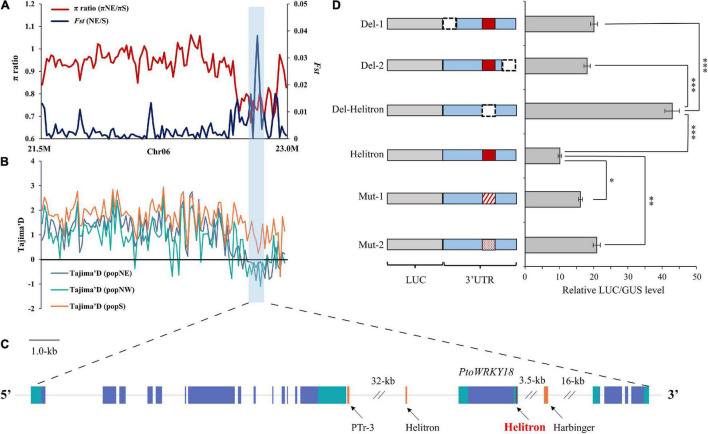
An adaptive TE within selection regions among the three *P. tomentosa* natural climatic regions. **(A–C)** Adaptive TEs with a selection signal between each pairwise comparison. The values of *F*_*st*_, π, and Tajima’s D were plotted using a sliding window of 500-bp. Target population (Orange), and reference population (blue). The horizontal dotted line represents the corresponding average value in all selected scanning areas. Genes at the bottom (blue rectangle, coding sequences; black line, introns and intergenic regions; green rectangle, 5′ and 3′ untranslated regions; red rectangle, Helitron insertion TEs; orange rectangle, other TEs). **(D)** Left panel, firefly luciferase reporter constructs fusing the full-length *WRKY18* 3′-UTR (Helitron), deletion variants (Del-1, Del-2, and Del-Helitron), and substitute variants (Mut-1, and Mut-2). Luciferase gene was dark gray with diagonal; 3′UTR is blue, the deleted area is represented by dotted line, and the variant is represented by red line with diagonal and point; The red rectangle in the 3′UTR represents the Helitron transposon. Right panel, relative luminous units and transcription levels quantified by qPCR (Helitron normalized to 1). The data in the right panel represent the mean ± standard deviation, *n* = 3. The *, ** and *** indicate significant difference at *P* < 0.05, *P* < 0.01, and *P* < 0.001, respectively (Student’s *t*-test). The data show the relative LUC/GUS level calculated by three independent replications.

Transposable element loci with selective advantage in a specific environment could spread in the population and accelerate its adaptation. Our aim was to identify the adaptive TE in different climatic regions of China (Supplementary Figure 7B). Then, we screened for adaptive TEs in sliding windows regions of each population (Supplementary Figures 6, 8A–C, Supplementary Data 15, and Supplementary Method 1). According to the first 5% of the empirical distribution of the logarithmic ratio (π_region_1_/π_region_2_) and the population differentiation Statistics (*F*_*st*_) value of each paired comparison between climatic regions, 4, 137, and 231 TE sites were obtained, respectively (Supplementary Data 16). Among TE sites, 1, 24, and 45 TE sites had significant low π values (2-kb around regions) in popNE, popNW, and popS, respectively (Supplementary Table 5), suggesting that the TE insertion alleles might be positive selection targets. In order to further confirm that the identified TEs are positive targets, we screened Tajima’s D values of SNP sites in 20-kb regions surrounding each candidate TE (10-kb upstream and 10-kb in downstream of each TE; Supplementary Method 1). Finally, two adaptive TE candidates having significantly higher or lower Tajima’s D values in their flanking 20-kb regions compared the target population with the reference population (Figures 5A–C and Supplementary Table 5), indicating that these two adaptive TEs showed higher haplotype homozygosity in TE insertion alleles than those without TEs.

As Figure 5 shown, an adaptive TE inserted 837-bp in 3′UTR of *PtoWRKY18* in sweep regions of chr6-22–23 Mb. To evaluate whether TE insertion had an effect on the expression level of *PtoWRKY18*, we transiently expressed the 3′UTR of *PtoWRKY18* in protoplasts using a dual luciferase reporter assay (Figure 5D). The results showed that the complete 3′UTR (Helitron) and the shortened 3′UTRs containing the Helitron (Del-1 and Del-2) significantly repressed luciferase expression level, but the 3′UTR lacking the Helitron (Del-Helitron) had no repression effect (Figure 5D). Constructs with mut-1 (an unrelated sequence) or mut-2 (another retrotransposon) showed an approximately 2-fold increase in the relative LUC/GUS level of Helitron compared to constructs with a complete 3′UTR (Figure 5D), confirming that the Helitron transposon repressed gene expression. Another Helitron DNA transposon insertion at the 3′UTR in accession #3601 is uniquely present in popNW, and harbored in the upstream of *PtoWRKY28*, which is preferentially expressed in flower bud and mature leaves, encoding a protein involving in defense response (Babitha et al., 2013; Chen et al., 2013; Supplementary Figures 9A–C).

## Discussion

### Transposable Elements as a Contributor of Regulatory Elements

A large number of studies have shown that TEs can directly affect the regulation of nearby gene expression in a variety of ways, including transcriptional level and post transcriptional level (Kobayashi et al., 2004; Studer et al., 2011; Chuong et al., 2017). Similarly with our study, gene expression increased with increasing distance from the nearest TE (Figure 2A), and the Helitron inserted into 3′UTR significantly repressed nearby gene expression (Figure 5D). However, these mechanisms were found in the laboratory by mutation analysis caused by single TE insertion (Studer et al., 2011; Sun et al., 2014). Actually, it is now clear that in the distant past, the same changes have taken place in gene structure and expression level, which have been preserved by natural selection (Brosius, 2003; Marino-Ramirez et al., 2005; Gonzalez et al., 2009). Jordan et al. (2003) reported that nearly 25% of the experimentally characterized human promoters contain TE derived sequences, including empirically defined *cis* regulatory elements. Further genome-wide analysis showed that there were many promoters in the human genome (Bousios et al., 2016) and mouse genes (van de Lagemaat et al., 2003) are derived from primate specific and rodent specific TE sequences, respectively. Hence, the insertion of these TEs may help to establish lineage specific patterns of gene expression (Marino-Ramirez et al., 2005). Further evidence that TEs generally obtained regulatory function comes from TE fragments that are highly conserved in mammals; however, whether TEs performed the same function in plants was rarely reported. In our study, The conserved TEs were overexpressed in the predicted *cis* regulatory module, that was, a genomic fragment containing a dense array of TFBSs; such as WBOXATNPR1, which related *WRKY18* was found at a high frequency. Therefore, we hypothesize that these elements tend to cluster around genes that were involved in development and biological pathways of transcriptional regulation. Approximately one-tenth of homologous genes, thousands of which were derived from conserved TEs, overlapped with stress-responsive *cis*-elements that regulated downstream functional genes, which implied that they provided promoter sequences or TFBS for regulatory elements (Supplementary Data 8). More and more highly conserved TEs had been recorded as transcriptional enhancers (Bejerano et al., 2006; Santangelo et al., 2007).

Whether regulatory elements produced *de novo* by some mutations or pre-existing in TE sequences, TEs had been a profuse source of new regulatory sequences. The dispersion of the extended TE family throughout the genome may allow the recruitment of the same regulatory motifs at many chromosomal locations and the introduction of multiple genes into the same regulatory network (Slotkin and Martienssen, 2007; Warnefors et al., 2010; Agren et al., 2014). Although our research suggested a correlation with potential adaptive significance, future research should explore whether TEs could drive adaptive evolution in population of woody plants and confer an adaptive advantage.

### Expression Levels of Transposable Elements, 24-nt siRNA and Adjacent Genes

Previous studies had showed that TEs in 3′UTRs could alter gene expression level *via* the regulation of methylation in *Arabidopsis* (Le et al., 2015; Li et al., 2018) and could change the translation efficiency in rice (Naito et al., 2009; Shen et al., 2017). Similarly, here we found that a TE insertion in the 3′UTR affected *WRKY18* expression level (Figure 5). In addition, the TEs and 24-nt siRNAs distributions were positively correlated with each other in *P. trichocarpa* (Figures 2, 3A), and it is essential to initiate and maintain DNA methylation during TE insertions (Feschotte et al., 2002; Liu et al., 2004; Jia et al., 2009; Casacuberta and Gonzalez, 2013). The gene expression level increased with the distance to the nearest TE, and this relationship was stronger when the TE closest to a gene was siRNA+ (Figure 2). The results of the expression model under heat stress were confirmed by comparing whether there were different genes in the presence of nearby TEs whether to be active under heat stress (Figure 2). It has been suggested that decreased gene expression is a direct consequence of TE insertion. Indeed, new insertions of a miniature inverted repeat transposable element (MITE) insertion-*mping* in rice actually enhanced gene expression (Naito et al., 2009), suggesting that the impact may vary by taxon, TE family and individual TE. Nonetheless, for all families shown in Figure 3A, the distribution tendency of TEs-24 nt siRNA+ were almost consistent (although not always significantly so), with the exception of Helitron transposons. Therefore, Helitron transposons made the most significant contribution to regulate nearby genes with 24-nt siRNAs targeted. Additionally, the relationship between TE proximity and gene expression level varied under high temperature treatment; in our analysis, gene expression under heat stress seems to be more sensitive to the proximity of TEs (Figure 2A). For example, it is not clear whether it reflects greater robustness of gene expression under normal conditions, or perhaps stresses could enhance TE activity. Further study should explore whether high temperature or other stresses affect gene stability by changing nearby TEs activity.

### Transposable Elements Promote Adaptive Evolution in Natural Population of *Populus*

Transposable elements are considered to be a rapid adaptation factor because they can produce rich genetic variation in a limited time (Le Rouzic et al., 2007) and can affect phenotypic variation (Martin et al., 2009). In our study, we discussed the interpretation of the evolution of transposable factors and their impact on the host genome from different angles. On the one hand, we investigated the transposon landscape in four poplar genomes and evaluated its dynamic characteristics. This gives us an in-depth understanding of the dynamics of transposon distribution and the selection force that forms the transposon pattern. On the other hand, we deeply studied a special case of poplar, that is, adaptive TEs insertion with selective advantage in a specific environment, which may spread in the population and accelerate the adaptation of organisms. TEs are the main source of genomic mutations and, like any environmental mutagen, occasionally lead to beneficial changes (Chuong et al., 2017). From the perspective of evolution, it is important to study two aspects of TEs, namely, their evolutionary dynamics in the genome and their contribution to adaptation in global climate change.

Because TEs are an important source of genetic variation, they can promote the evolution of organisms in many ways, such as obtaining coding ability and changing coding sequence (Finnegan, 1989; Marino-Ramirez et al., 2005; Agren et al., 2014), and effecting the gene expression level (Wessler et al., 1995; Cui and Fedoroff, 2002; Studer et al., 2011). Specially, TE insertions or mutations may affect adaptation to the environment (Aravin et al., 2007; Joly-Lopez et al., 2012; Casacuberta and Gonzalez, 2013), and TE insertions or mutations that had beneficial effects on the adaptation of natural populations might become fixed. In our study, we used a representative *P. tomentosa* population which distributed a wide geographical area of northern China (30°N–40°N, 105°E–125°E) to explore the relationship between genetic diversity and adaptive evolution. However, the number of natural populations had decreased significantly, mainly due to environmental change, would further threat to the genetic resources. In this study, we found a Helitron TE locus probably become a target of positive choice and therefore contribute to adaptation of *P. tomentosa*. Consistent with that, TEs in 3′UTRs can change gene expression *via* regulating DNA methylation in *Arabidopsis* (Saze and Kakutani, 2014) and can decrease translation efficiency in rice (Shen et al., 2017). In addition, a Helitron-induced RabGDIα variant could cause quantitative recessive resistance to maize rough dwarf disease (Liu et al., 2020). Other studies also found Helitrons, upon heat shock, upregulated of nearby genes in *Caenorhabditis elegans* and promoted adaption on heat environment (Garrigues et al., 2019). Future researches could explore whether this phenotypic diversity will eventually bring adaptation advantages, and whether TEs could promote adaptation to climate change.

## Data Availability Statement

The original contributions presented in the study are publicly available. This data can be found here: Raw data of RNA-seq and resequencing are available for download at the BIGD Genome Sequence Archive (https://bigd.big.ac.cn) under accession numbers CRA001776 and CRA000903, respectively.

## Author Contributions

DZ designed the experiments, obtained funding, and was responsible for this article. YZ collected and analyzed the data and wrote the manuscript. YZ, XL, JX, WX, SC, XZ, SL, and JW performed the experiments. YE-K revised the manuscript and provided valuable suggestions to the manuscript. All authors read and approved the manuscript.

## Conflict of Interest

The authors declare that the research was conducted in the absence of any commercial or financial relationships that could be construed as a potential conflict of interest.

## Publisher’s Note

All claims expressed in this article are solely those of the authors and do not necessarily represent those of their affiliated organizations, or those of the publisher, the editors and the reviewers. Any product that may be evaluated in this article, or claim that may be made by its manufacturer, is not guaranteed or endorsed by the publisher.

## Footnotes

1
http://www.repeatmasker.org

2
https://bigd.big.ac.cn

## Supplementary Material

Supplementary Figure 1 Transposons and genes show negative correlation distribution on *P. trichocarpa* chromosomes.

Supplementary Figure 2 Transposons distributed on *P. trichocarpa* chromosomes.

Supplementary Figure 3 Distribution of TEs in four *Populus* genomes.

Supplementary Figure 4 GO-term analysis of genes with transposons.

Supplementary Figure 5 Six regulatory motifs significantly enriched in the TEs drive promoter regions.

Supplementary Figure 6 Flowchart of the polymorphic TE.

Supplementary Figure 7 Diversity in *P. tomentosa* population.

Supplementary Figure 8 Adaptive selection signals within polymorphic TE insertions among the three *P. tomentosa* natural climatic regions.

Supplementary Figure 9 A adaptive TE within selection regions among the three *P. tomentosa* natural climatic regions.

Supplementary Method 1 Supplementary methods for this study.
