# Supplementary figures and images for: Transposable Elements: Distribution, Polymorphism, and Climate Adaptation in Populus
Source: Front Plant Sci. 2022 Feb 1;13:814718. doi: 10.3389/fpls.2022.814718 (PMC8843856; doi:10.3389/fpls.2022.814718)

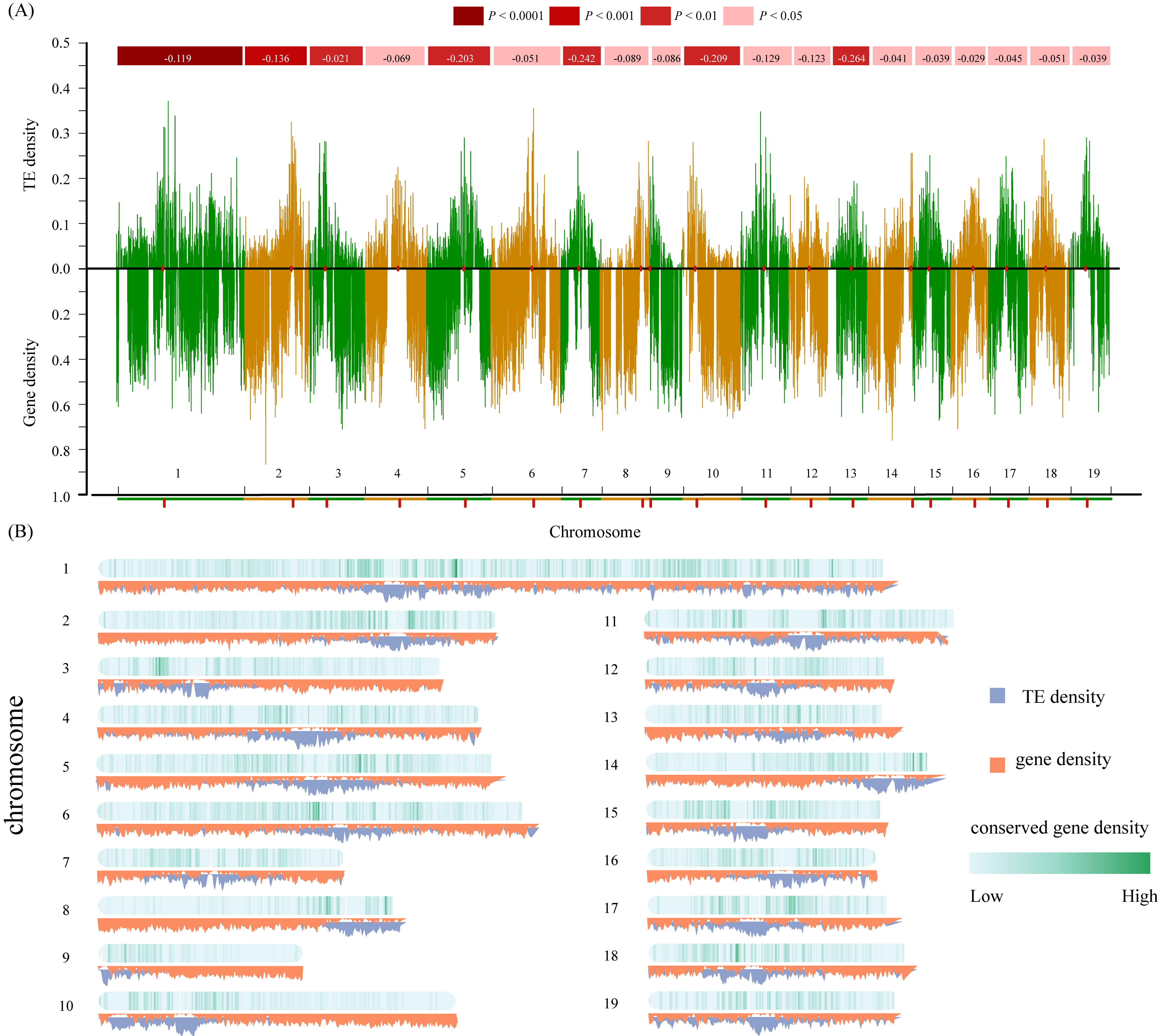

Supplement: Supplementary Figure 1 — Transposons and genes show negative correlation distribution on P. trichocarpa chromosomes. [file Image_1.TIF]

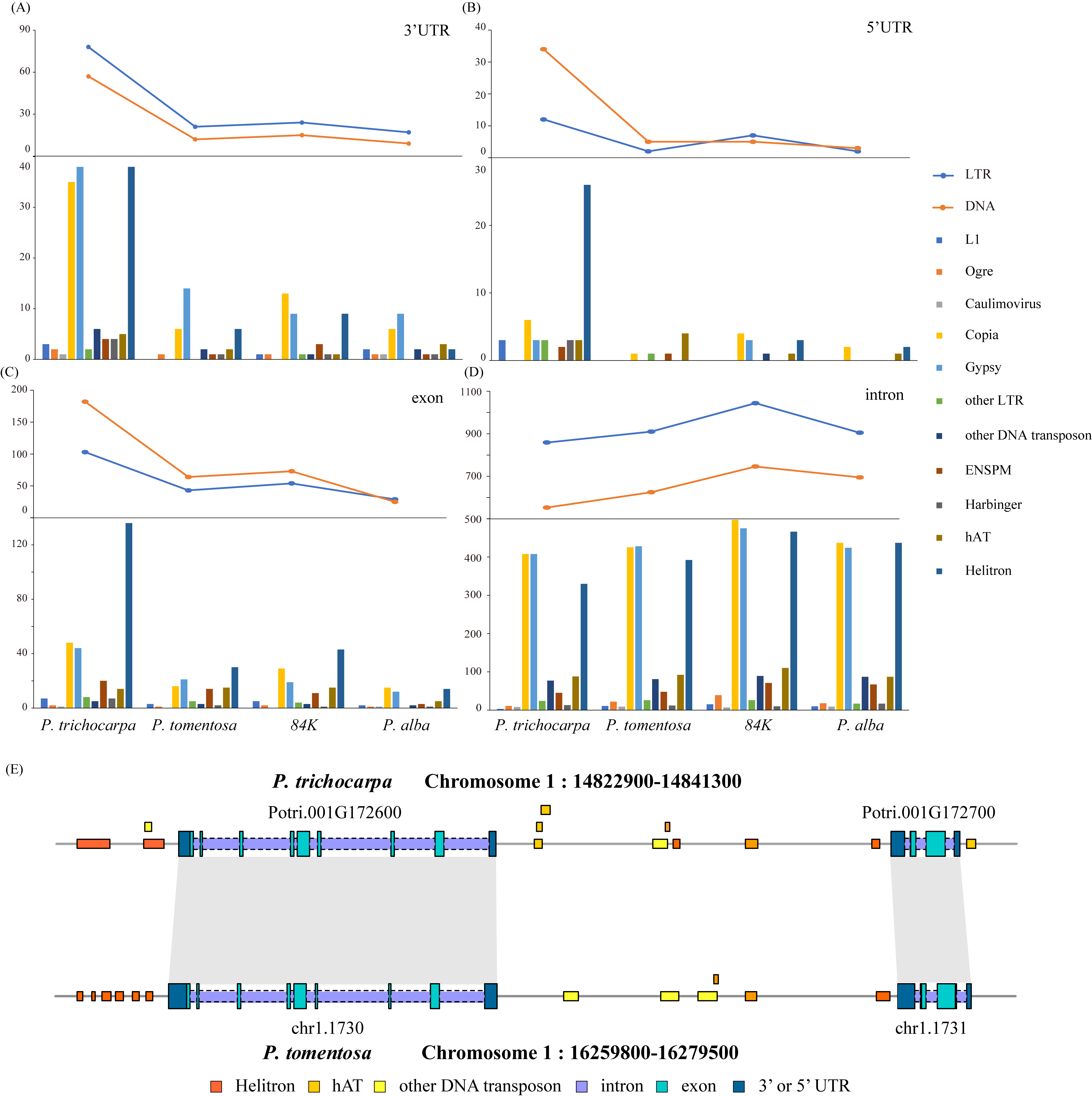

Supplement: Supplementary Figure 2 — Transposons distributed on P. trichocarpa chromosomes. [file Image_2.TIF]

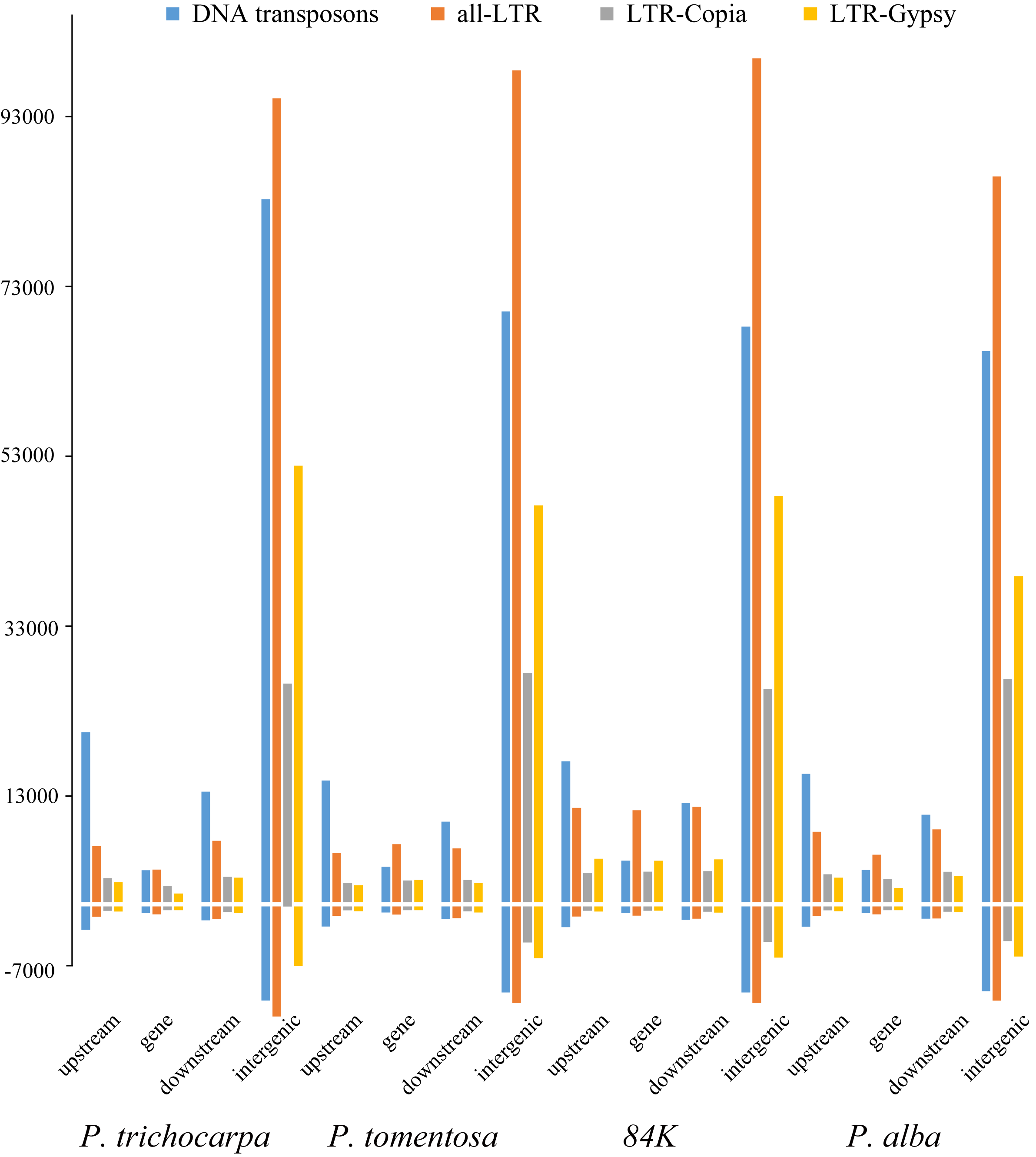

Supplement: Supplementary Figure 3 — Distribution of TEs in four Populus genomes. [file Image_3.TIF]

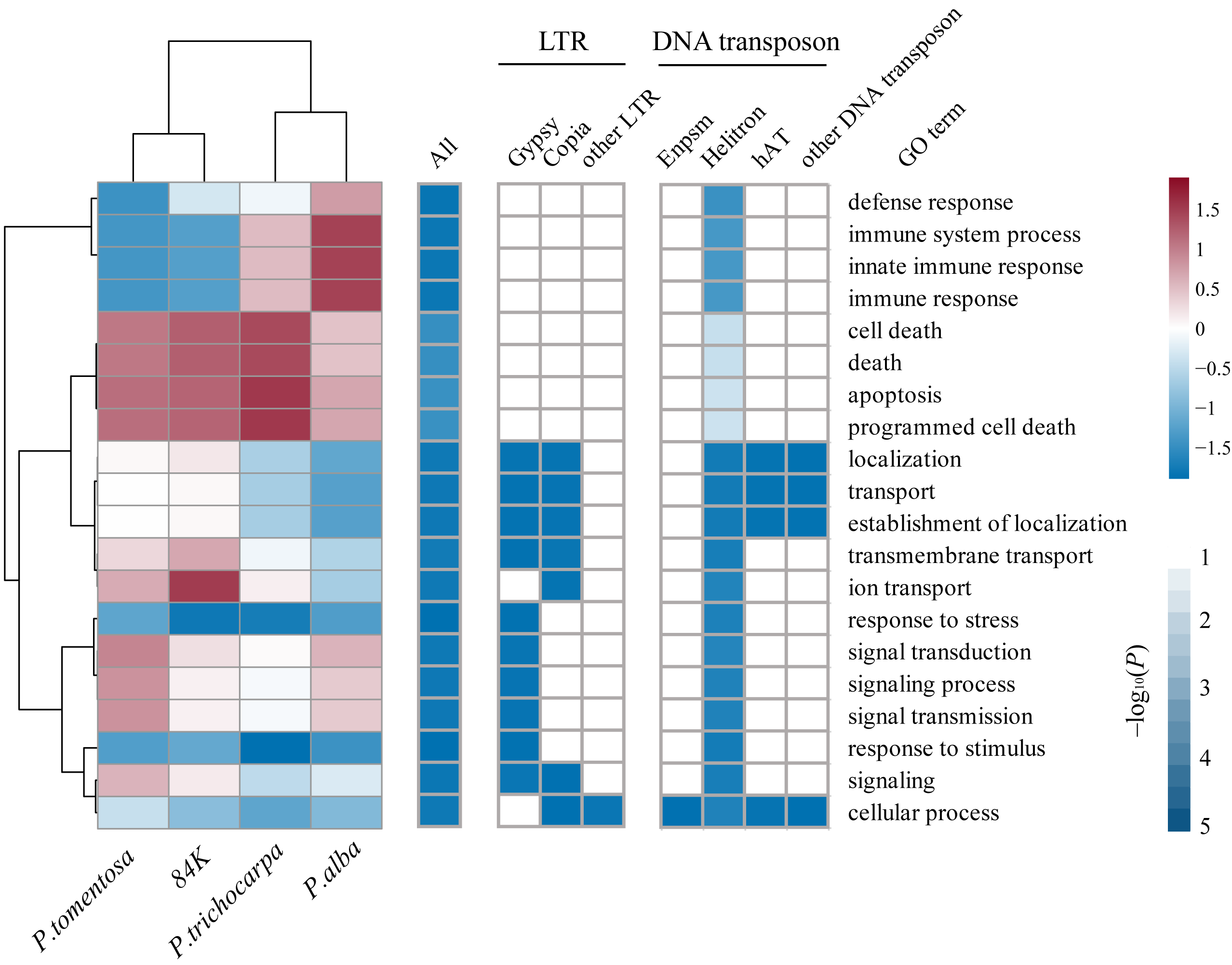

Supplement: Supplementary Figure 4 — GO-term analysis of genes with transposons. [file Image_4.TIF]

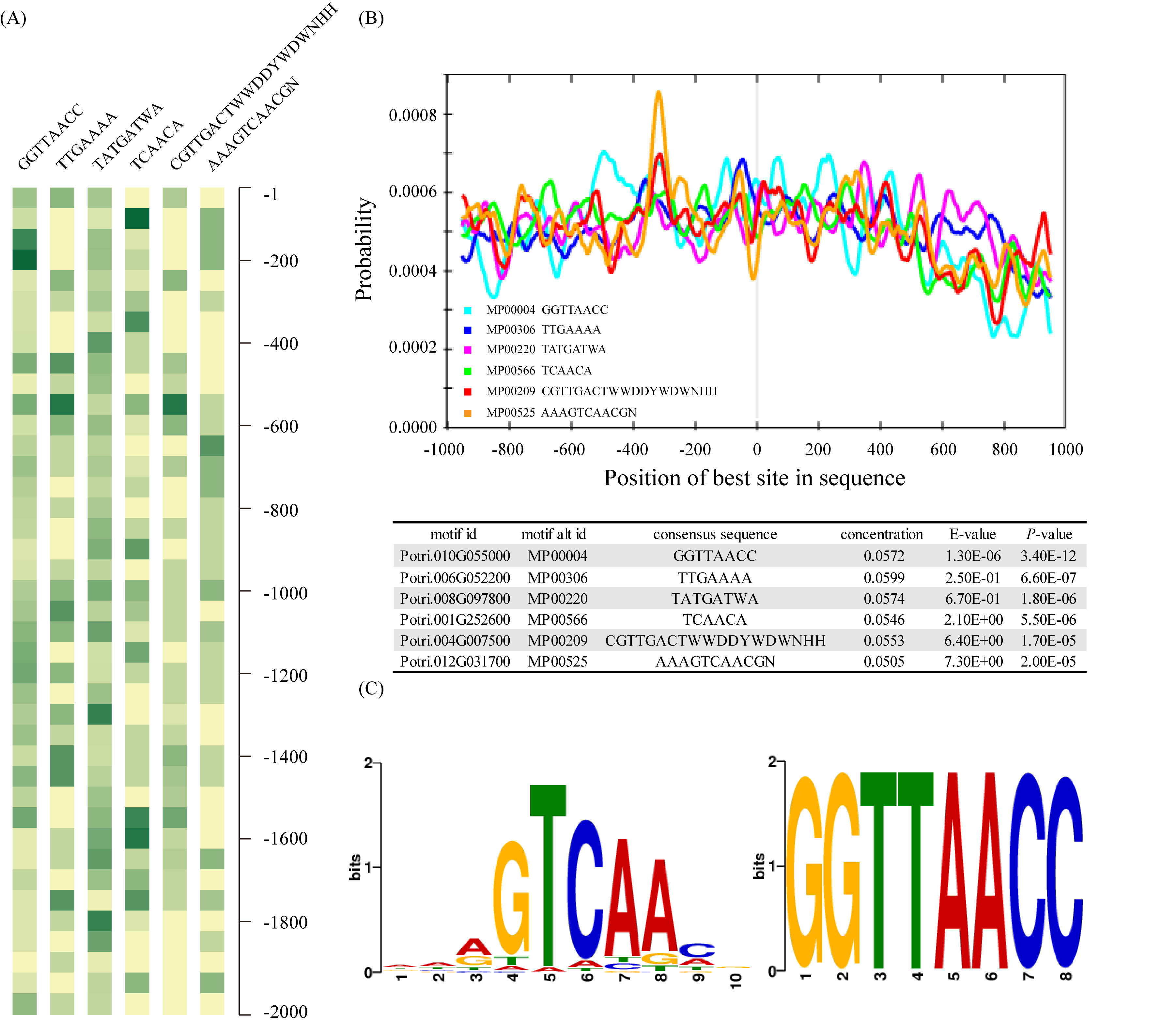

Supplement: Supplementary Figure 5 — Six regulatory motifs significantly enriched in the TEs drive promoter regions. [file Image_5.TIF]

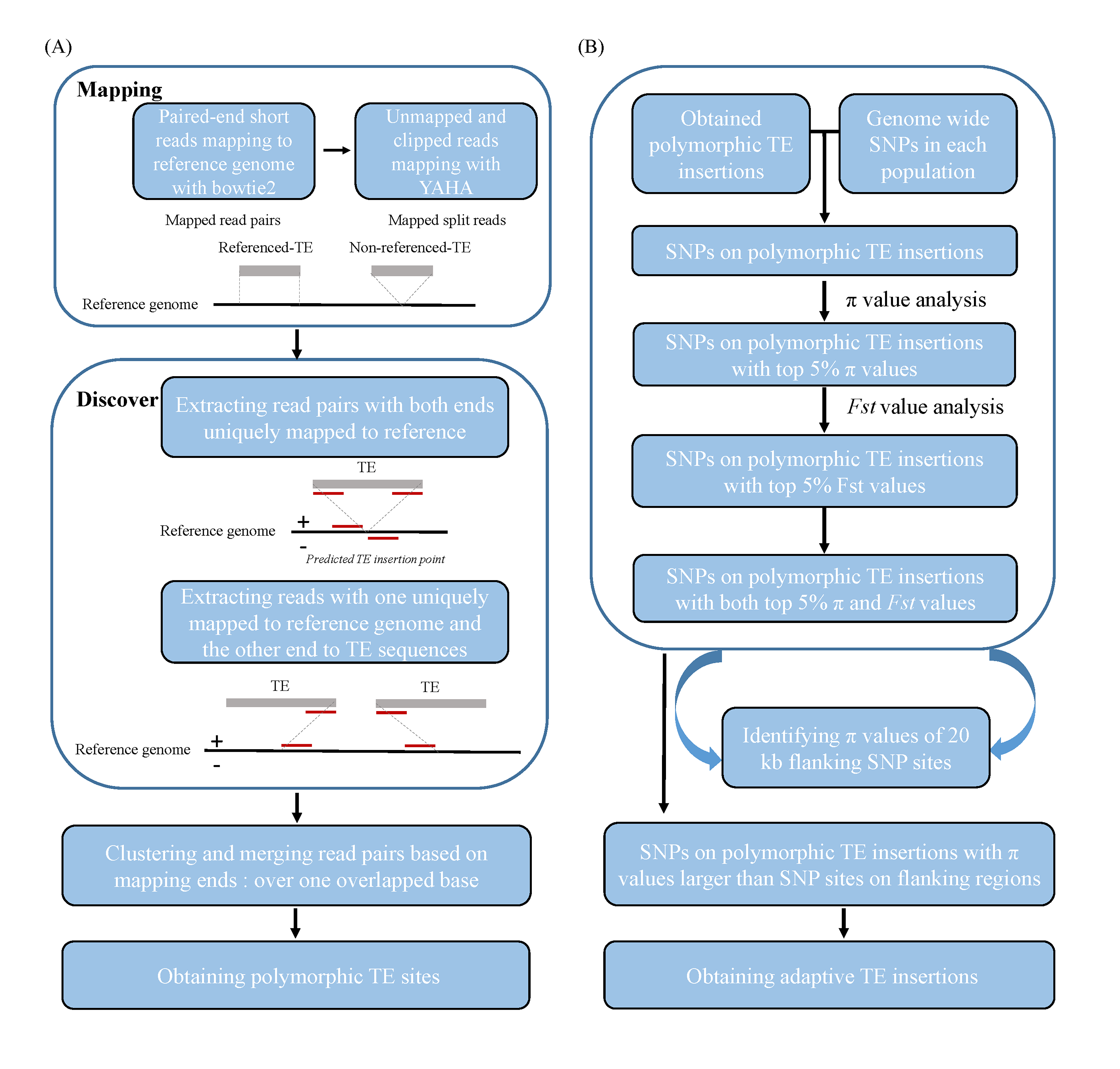

Supplement: Supplementary Figure 6 — Flowchart of the polymorphic TE. [file Image_6.TIF]

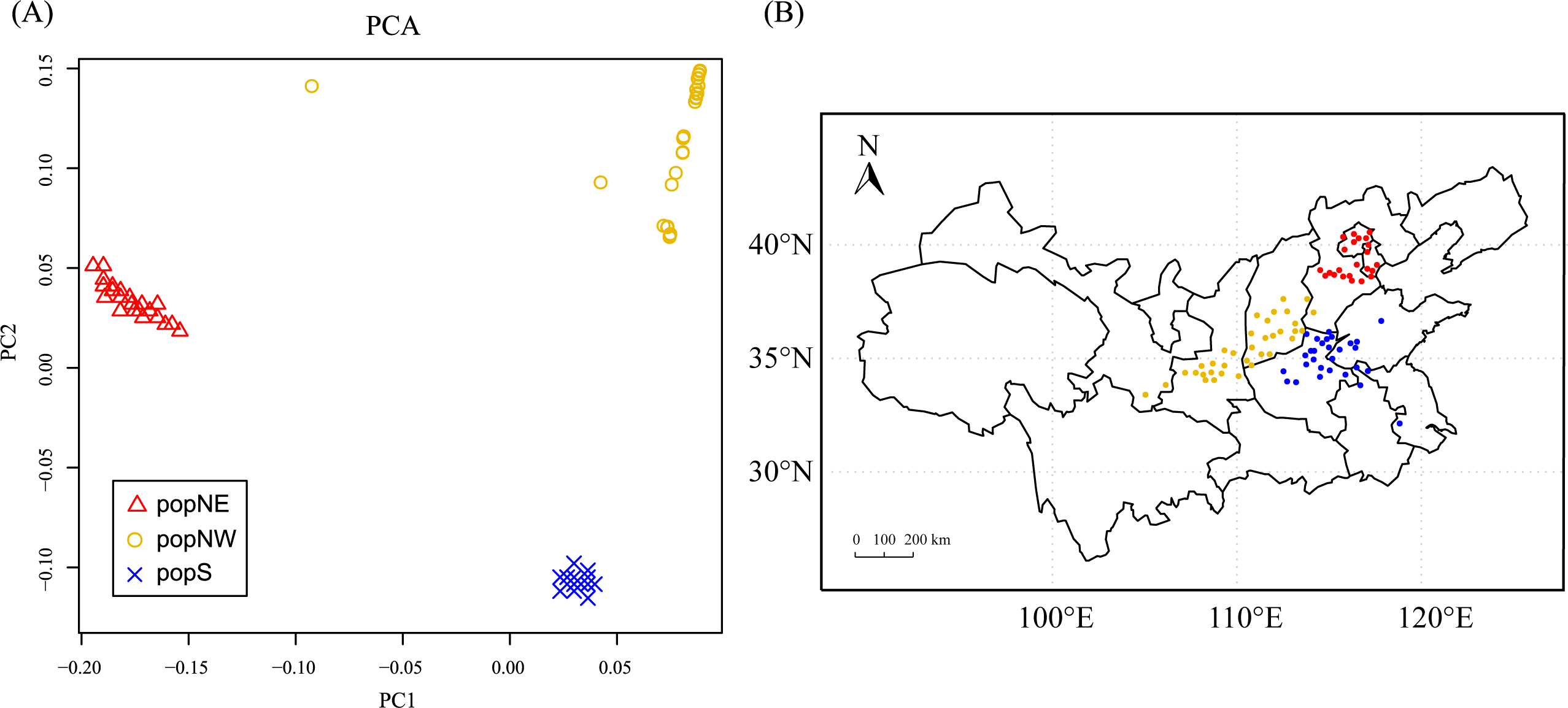

Supplement: Supplementary Figure 7 — Diversity in P. tomentosa population. [file Image_7.TIF]

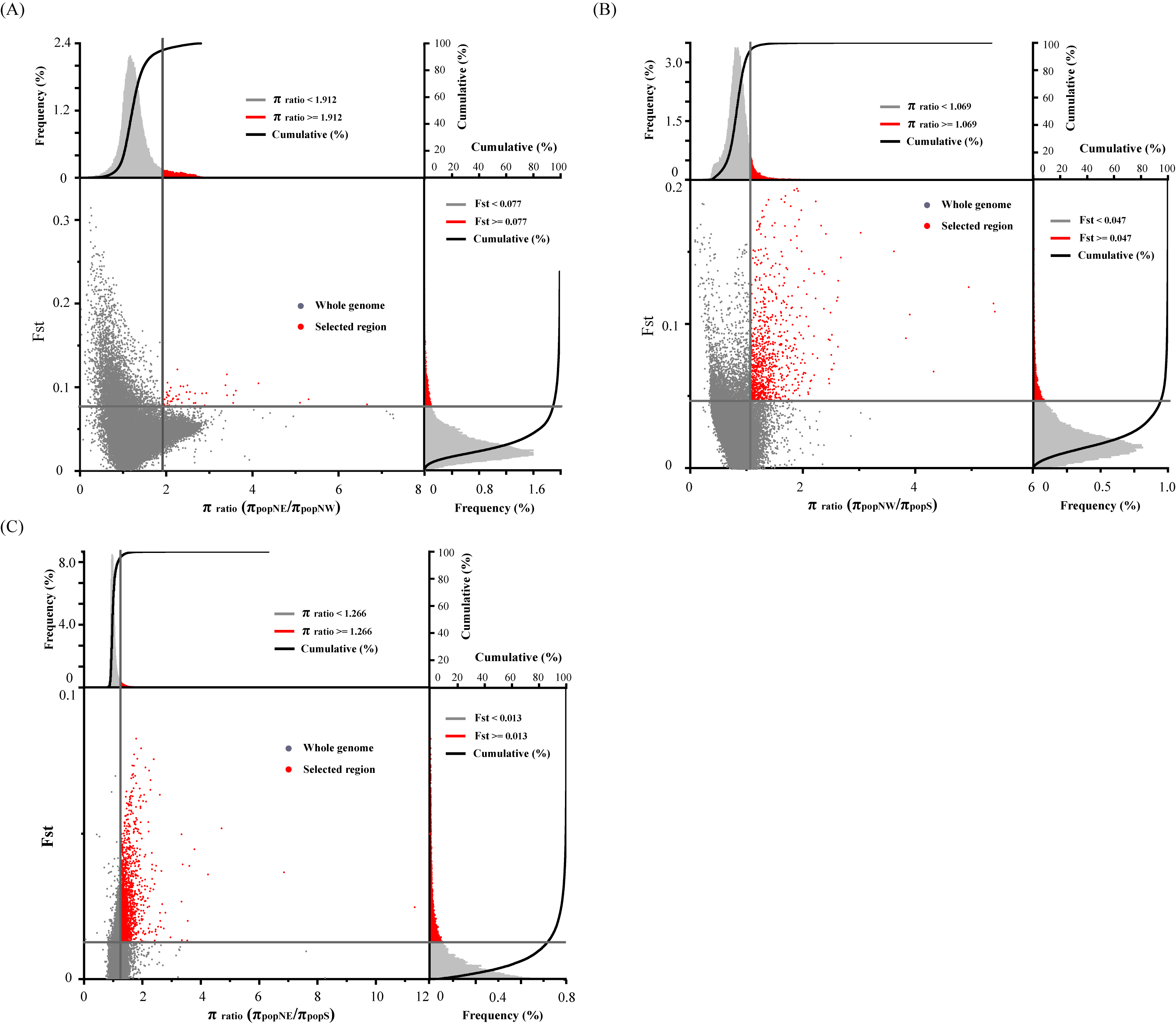

Supplement: Supplementary Figure 8 — Adaptive selection signals within polymorphic TE insertions among the three P. tomentosa natural climatic regions. [file Image_8.TIF]

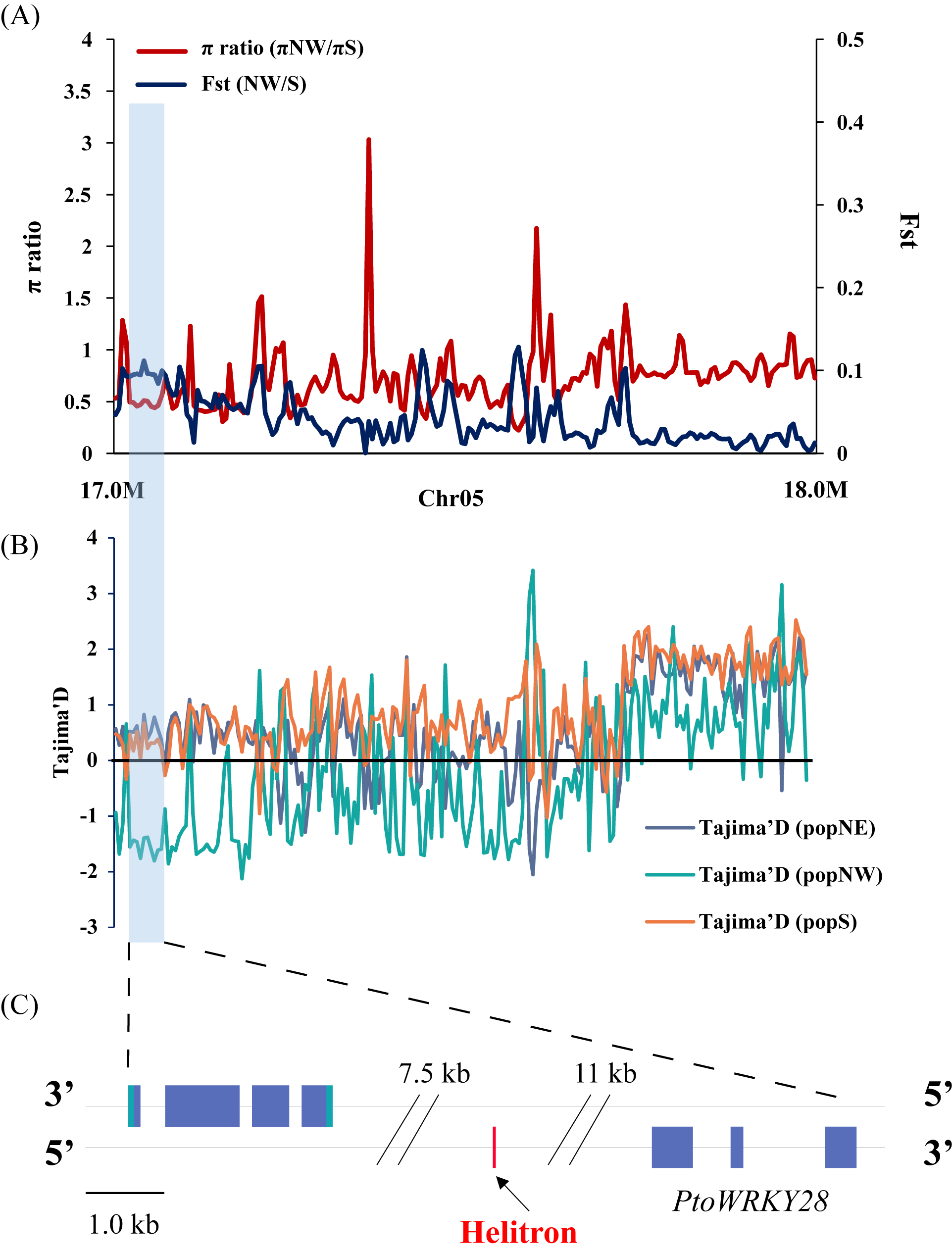

Supplement: Supplementary Figure 9 — A adaptive TE within selection regions among the three P. tomentosa natural climatic regions. [file Image_9.TIF]
