# Supplementary material for: Transposable Elements: Distribution, Polymorphism, and Climate Adaptation in Populus
Source: Front Plant Sci. 2022 Feb 1;13:814718. doi: 10.3389/fpls.2022.814718 (PMC8843856; doi:10.3389/fpls.2022.814718)
Supplement: Supplementary Method 1 — Supplementary methods for this study. [file Table_8.DOCX]

**Supplementary data information**

Article title: Transposable elements: distribution, polymorphism, and climate adaptation in *Populus*

**The following are Supplementary to this article:**

**Method S1.** Supplementary methods for this study.

**Figure S1.** Transposons and genes show negative correlation distribution on *P. trichocarpa* chromosomes*.*

**Figure S2.** Transposons distributed on *P. trichocarpa* chromosomes.

**Figure S3.** Distribution of TEs in four *Populus* genomes.

**Figure S4.** GO-term analysis of genes with transposons.

**Figure S5.** Six regulatory motifs significantly enriched in the TEs drive promoter regions.

**Figure S6.** Flowchart of the polymorphic TE.

**Figure S7.** Diversity in *P. tomentosa* population.

**Figure S8.** Adaptive selection signals within polymorphic TE insertions among the three *P. tomentosa* natural climatic regions.

**Figure S9.** A adaptive TE within selection regions among the three *P. tomentosa* natural climatic regions.

**Table S1.** Classification of transposable elements in the *Populus trichocarpa*, *P. tomentosa*, *P. alba × P. glandulosa* (84K), and *P*. *alba genome.* ^a)^

**Table S2.** The published re-sequencing genomes of *P. tomentosa* population used in this study.

**Table S3.** The numbers of TE loci in the tree *P. tomentosa* populations.

**Table S4.** Composition of polymorphic TEs in different populations.

**Table S5.** Adaptive TE candidates in our study.

**Table S6.** TEs populations validation.

**Table S7.** Primers used in this study.

**Data S1.** 21,862 collinear genes across four *Populu*s species, *P. trichocarpa*, *P. tomentosa*, *P. alba × P. glandulosa* (84K), and *P. alba*.

**Data S2.** 25,321 collinear TEs across four *Populu*s species, *P. trichocarpa*, *P. tomentosa*, *P. alba × P. glandulosa* (84K), and *P. alba*.

**Data S3.** Distribution of collinear TEs in related genomic regions in *P. trichocarpa*.

**Data S4.** Distribution of collinear TEs in related genomic regions in *P. tomentosa*.

**Data S5.** Distribution of collinear TEs in related genomic regions in *P. alba × P. glandulosa* (84K).

**Data S6.** Distribution of collinear TEs in related genomic regions in *P. alba*.

**Data S7.** GO terms of motifs identified by GOMo^a)^ in *P. trichocarpa*.

**Data S8.** Enriched regulatory elements acting in 2-kb upstream regions of genes with conserved TEs in *P. trichocarpa*.

**Data S9.** All the TF binding sites in 2-kb upstream regions with conserved TEs in *P. trichocarpa*.

**Data S10.** Polymorphic TEs of *P. tomentosa* population.

**Data S11.** Polymorphic TEs of popNE.

**Data S12.** Polymorphic TEs of popNW.

**Data S13.** Polymorphic TEs of popS.

**Data S14.** Polymorphic TEs present in the CDS regions of genes.

**Data S15.** Compared *F_st_* values and π values among three subpopulutions of *P. tomentosa*.

**Data S16.** Polymorphic TE sites harbored on 60 (popNE vs. popNW), 923 (popNW vs. popS), and 1717 (popNE vs. popS) significant sliding windows with the top 5% highest log-ratio and *Fst* values.

**Method S1**

**TE annotation and polymorphic TE sites identification**

For each genome, RepeatModeler (v2.0.1) was used for the de novo identification of TEs ([Wicker et al., 2007](#_ENREF_27)), and LTR-retriever (v2.8) was used to identify *de novo* LTR retrotransposons ([Ou and Jiang, 2018](#_ENREF_20)), which integrated LTRharvest from the Genome Tools v1.5.9 package ([Ellinghaus et al., 2008](#_ENREF_8)), LTRfinder ([Xu and Wang, 2007](#_ENREF_29)), and MGEScan-LTR ([Estill and Bennetzen, 2009](#_ENREF_9)). The *de novo* annotation of TEs using RepeatModeler v2.0.1 with the parameters “ncbi” and “LTRStruct” to generate TE family consensus sequences. We then excluded unknown TE consensus sequences by TEclass v2.1.3 with default parameters ([Abrusan et al., 2009](#_ENREF_1)). Remaining consensus sequences that were assigned to defined TE superfamilies were used in the next steps. To reduce false positives, unclassified repeats were compared with annotated genes and eliminated if they exhibited over 80% identity to annotated genes over 80 bp, as described in previous study ([Niu et al., 2019](#_ENREF_18)). The remaining predictions were grouped if >= 80% identical over >= 80% of the aligned sequence ([Hollister et al., 2011](#_ENREF_11)). TEs in the reference genomes of *P. trichocarpa, P. tomentosa, P. alba* × *P. glandulosa* (84K), *P. alba* were annotated using RepeatMasker v4.0.6 (no_is -pa 30 -species *Populus* -s -nolow -norna; <https://www.repeatmasker.org>). The identified repeats were appended to RepBase (RM Database; Version: 2017-01-27) and Tandem Repeat Finder (TRF; v4.09), resulting to be annotated by RepeatMasker (v4.0.6).

The polymorphic TEs were identified using TEPID ([Stuart et al., 2016](#_ENREF_22)) based on the raw reads of 87 resequencing genomes of *P. tomentosa* (Tian et al., 2016). The polymorphic TE sites are TE loci in which some accessions harbor TE insertions but others do not.

Intact LTR retrotransposons of 18 different species (Figure 1C) were identified with LTR-retriever v2.8 ([Ou and Jiang, 2018](#_ENREF_20)), using default parameters, and LTRs were aligned using MUSCLE v3.8.31 ([Edgar, 2004](#_ENREF_7)). The distance *k* between the two LTRs or a retrotransposon was calculated with the Kimura two-parameter model ([Kimura, 1980](#_ENREF_13)). The insertion time of an element was calculated as *k*/2*m*, where m is the rate of nucleotide substitution based on the observed mutation rate of 7 × 10^−9^ per site per generation (assumed to equal 1 year) ([Ossowski et al., 2010](#_ENREF_19)).

**sRNA processing and identification**

The sequences generated from the leaves exposed to heat stress treatments were used to detect the transcript abundance of mature sRNAs. All sRNA reads, referred to as raw reads, were processed to remove adaptors, low-quality tags and contaminants. Clean reads were then mapped to version 4.1 of *P. trichocarpa* genome with no more than one mismatch. These perfectly aligned sequences were annotated by BLAST-searching them against the GenBank and Rfam databases (version 13; http://rfam.xfam.org/), allowing one mismatch. The tRNAs, rRNAs, snRNAs, snoRNAs, and scRNAs were removed from the sequencing reads. The remaining unannotated sRNAs were searched against the known miRNAs from miRBase version 22.1 (http://www.mirbase.org/), allowing a maximum of two mismatches. Then, remaining unannotated unique sequences were mapped to the *P. trichocarpa* genome to uncover novel miRNAs from poplar, according to the established criteria (Meyers 2008), using MiReap software (<https://sourceforge.net/projects/mireap/>).

**Identification of syntenic genes/TEs among four poplar species**

To identify orthologous genes/TEs among *P. trichocarpa, P. tomentosa, P. alba* × *P. glandulosa* (84K), *P. alba* genomes, MCScanX ([Wang et al., 2012](#_ENREF_26)) was used to determine the synteny of homologous protein-encoding genes/TEs among the four species. Protein and transposons sequences of each species were used as queries to search against the corresponding genome sequence using BLAST v2.0.9+ ([Camacho et al., 2009](#_ENREF_4)). Then the syntenic genes/TEs were excluded from the total data set by MCScanX ([Wang et al., 2012](#_ENREF_26)), and the remaining genes/TEs were defined as non-syntenic genes. The values of synonymous (*K_s_*), non-synonymous (*K_a_*) substitution rates and the *K_a_*/*K_s_* ratio were estimated for each collinear gene pair using codeml in PAML v4.9 ([Yang, 2007](#_ENREF_30)). Transversion rate at 4DTV for each collinear gene pair was calculated based on 4-fold degenerate sites following the HKY substitution model.

**Estimating the abundance of TE insertions**

We mapped 87 resequenced genomes of *P. tomentosa* individuals (Table S2) to the respective TE-merged-reference with bwa (v0.7.17) ([Li and Durbin, 2009](#_ENREF_15)) using the bwa-sw algorithm ([Li and Durbin, 2010](#_ENREF_16)). We estimated the abundance of TE insertions with PoPoolation TE similarly as described by Kofler et al. ([Kofler et al., 2012](#_ENREF_14)) using the following settings: identify-te-insertions.pl –te-hierarchy-level family, –min-count 3, –min-map-qual 15, –narrow-range 100; crosslink-tesites.pl –min-dist 85, –max-dist 300; estimate-polymorphism.pl –te-hierarchy-level family, –min-map-qual 15; Subsequently, we iteratively subsampled repeated TE identification with PoPoolation TE, until we obtained similar physical coverages in all populations.

**Genes’ expression of *P. trichocarpa* under heat stress**

Leaves were harvested from 1-year-old *P. trichrocarpa* at 0, 4, 8, 12, 24, 36, and 48 hours after exposure to 40°C heat treatment. The completely expanded leaves were collected, immediately immersed in liquid nitrogen, and stored at –80°C until further use. Total RNA was extracted according to the modified hexadecyl trimethyl ammonium bromide (CTAB) assay described by Chen et al. ([Chen et al., 2004](#_ENREF_5)); to be specific, isopropanol rather than lithium chloride was used for RNA precipitation, and the genomic DNA was eliminated using DNase. The quality of RNA was evaluated using an Agilent Bioanalyzer 2100 (Agilent Technologies, Santa Clara, CA, USA), Qubit 2.0 Fluorometer (Invitrogen, Life Technologies, CA, USA), and NanoDrop ND-2000 (Thermo Fisher Scientific, Waltham, MA, USA). Afterwards, high-quality RNA was used to build the cDNA library in accordance with TruSeq RNA Sample preparation instructions (Illumina, San Diego, CA, USA).

Following analysis, those samples that had the OD 260/280 ratio of 1.9–2.1 and the OD 260/230 ratio of 1.8–2.2 were selected for subsequent use. Paired-end sequencing was performed by Novogene (Beijing, China) on an Illumina HiSeq 4000 platform (Illumina), generating 150-bp paired-end reads using TruSeqPEClusterKitv3-cBot-HS (Illumina) according to the manufacturer’s instructions and the strand-specific libraries were sequenced. The raw RNA-Seq data were quality-filtered using fastp software with default parameters (Chen et al. 2018). Quality control of raw reads was carried out as the following process, including: (1) removing paired reads with adapters (2) removing paired reads when the N content in any sequencing read > 10% of the read bases. (3) removing paired reads containing > 50% low-quality nucleotides (sQ <= 5). Clean reads were mapped to the v4.1 genome of *P. trichocarpa* using Hisat2 (v2.0.9) with the parameter -q -x -S -p. to generate read alignments for each sample ([Kim et al., 2019](#_ENREF_12)). To identify differently expressed genes (DEGs) in poplar in response to heat stress at different time points, a two-step analysis was carried out. First, fragments per kilobase per million mapped reads (FPKM) values were calculated to measure the abundance and expression of each transcript using Cufflinks v2.0.2 ([Trapnell et al., 2012](#_ENREF_24)). Second, differences in gene expression among samples were detected using Cuffdiff. Cuffdiff was used to identify up- and down-regulated genes by comparing the expression levels of transcripts under two conditions. The DEGs identified from the seven time-courses were filtered using the parameters *P*-value <= 0.05 and log_2_ fold change >= 1 (up-regulated genes) or <= -1 (down-regulated genes) to judge significant differences in gene expression. Then, the correlations among biological replicates were examined by computing the Spearman correlation coefficient.

**Detection of adaptive TE insertions**

The average number of pairwise differences per site between any two sequences, π ([Nei, 1987](#_ENREF_17)) was used for nucleotide diversity calculations. Nucleotide diversity (Nei's π, the average number of nucleotide differences per site between two DNA sequences chosen randomly from the sample population; Tajima F, 1983) was calculated using VCFtools with parameter ‘*--window-π*’ ([Danecek et al., 2011](#_ENREF_6)).

$$\text{π =}\sum_{ij} x_{i}x_{j}\pi_{ij}=2*\sum_{i=2}^{n} \sum_{j=1}^{i-1} x_{i}x_{j}\pi_{ij}$$

The population-differentiation statistic (*F_st_*) was measured for each pairwise region differentiation based on genetic polymorphism data ([Danecek et al., 2011](#_ENREF_6)). *F_st_* was calculated using VCFtools with parameters ‘--weir-fst-pop --fst-window-size --fst-window-step’ ([Danecek et al., 2011](#_ENREF_6)). The *F_st_* is a measure of population differentiation estimated from the average pairwise differences between variant in each analysis panel compared to the combined samples as described in International HapMap Consortium ([Barrett et al., 2005a](#_ENREF_2)). We used the top 5% of the empirical distribution of *F_st_* and π values within polymorphic regions as candidates representing signatures of significant divergent selection between each polymorphic TE region of subpopulations (popNE vs popNW, popNE vs. popS and popNW vs. popS), in which the latter was the reference population and the former was the object population. Selection sweeps were evaluated using an adjusted method for reduction of diversity (ROD) statistics according to Xu et al. ([Xu et al., 2011](#_ENREF_28)), by using 250-bp non-overlapping windows and an extensive 10-kb region upstream and downstream of each polymorphic TE region. All polymorphic TEs in the selective sweep regions were identified as putative adaptive TE insertions under selection.

In π statistical analysis, biallelic homozygous SNP sites and polymorphic TE loci with minor allele frequencies greater than 0.05 were used to calculate by VCFtools ([Danecek et al., 2011](#_ENREF_6)). The specific value of π between two subpopulations was used to detect selective sweeps. A significant high π ratio value is an indicator of the location of a TE locus in a selective sweep region. To estimate the significance of observed π values, we computed π values for TEs with the top 5% highest π values.

The *F_st_* statistic was estimated according to the method reported in a previous study ([Gonzalez et al., 2009](#_ENREF_10)). A significant high *F_st_* (top 5%) value is an indicator of positive selection on TE insertion alleles, with a significant higher π ratio value simultaneously. Finally, Tajima’s D value was also estimated for all SNPs in 20 kb regions flanking the candidate adaptive TEs. A significant higher Tajima’s D value for one candidate of one subpopulation (> 0); meanwhile the values of another two subpopulations were lower than 0; In another way, one subpopulation was < 0 and another two were > 0. This is an indicator of positive or negative selection on TE insertion alleles. Finally, the reserved TEs were identified as adaptive polymorphic TEs.

**Statistics formulas for *F_st_*, π and Tajima’s D calculations**

*F_st_* was calculated using VCFtools with parameters ‘*--weir-fst-pop--fst-window-size --fst-window-step*’. The *F_st_* is a measure of population differentiation estimated from the average pairwise differences between variant in each analysis panel compared to the combined samples as described in International HapMap Consortium ([Barrett et al., 2005b](#_ENREF_3)).

Tajima’s D ([Tajima, 1989](#_ENREF_23)) was calculated using VCFtools with parameter ‘*--TajimaD*’. Tajima’s D is computed as the difference between two measures of genetic diversity: the mean number of pairwise differences and the number of segregating sites, each scaled so that they are expected to be the same in a neutrally evolving population of constant size.

$$D=\frac{d}{\sqrt{{}_{V}^{^}{(d)}}}$$

*D* is calculated by taking the difference between the two estimates of the population genetics parameter theta. This difference is called *d*, and *D* is calculated by dividing *d*, by the square root of its variance (its standard deviation, by definition).$\sqrt{{}_{V}^{^}{(d)}}$

Nucleotide diversity {(Nei's π, the average number of nucleotide differences per site between two DNA sequences chosen randomly from the sample population; ([Nei, 1987](#_ENREF_17))} was calculated using VCFtools with parameter ‘*--window-π*’.

$$\text{π =}\sum_{ij} x_{i}x_{j}\pi_{ij}=2*\sum_{i=2}^{n} \sum_{j=1}^{i-1} x_{i}x_{j}\pi_{ij}$$

where X_i_ and X_j_ are the respective frequencies of the i^th^ and j^th^ sequences, π_ij_ is the number of nucleotide differences per nucleotide site between the i^th^ and j^th^ sequences, and n is the number of sequences in the sample.

**Transient LUC activity assays**

A full-length DNA fragment of *WRKY18* was amplified from *P. tomentosa* clone “1316”, and the full-length *WRKY18* without the 3'UTR was amplified from the WRKY18 full-length fragment with the gene sequence specific primers (Table S7). The full-length *WRKY18* without the Helitron was generated via fusion PCR using the primers listed in Table S7. All amplified fragments were cloned into pCAMBIA1301H, where the *WRKY18* genes were driven by its promoter region (2-kb fragments of upstream of the translational start site). The *WRKY18* 3'UTR deletion mutants (Del1, Del2) were generated from the full-length *WRKY18* 3'UTR with the primers. To replace the Helitron with other DNA fragments, a fragment from the 3'UTR was amplified with the GUS forward and reverse primers, and a Ty3/gypsy retrotransposon was amplified with the GUS forward and reverse primers from the “1316” clone genomic DNA. The *WRKY18* 3'UTR and pCAMBIA1301H fragments were amplified with the specific forward and reverse primers. The replaced fragments were substituted through blunt-end ligation between the *WRKY18* 3'UTR and pCAMBIA1301H fragments. Primers used for reporter gene constructs are shown in Table S7. Protoplast preparation of Arabidopsis and transient expression assays were performed as described previously ([Yoo et al., 2007](#_ENREF_31)). Briefly, each obtained reporter plasmid and 35S:GUS internal control were co-transformed into protoplasts ([Wang et al., 2014](#_ENREF_25);[Shen et al., 2017](#_ENREF_21)). For LUC assays, plastid combinations of various N- and C-terminal LUC fusions were co-transformed with the 35S:GUS internal control. The protoplasts were pelleted and resuspended in 1X cell culture lysis reagent (Promega). The GUS fluorescence was measured using a Modulus luminometer/fluorometer with a UV fluorescence optical kit (Promega). The LUC activity was detected with a luminescence kit using LUC assay substrate (Promega). The relative expression levels of reporter gene were defined as the LUC/GUS ratios. Each assay was performed three times independently in protoplasts.

**References**

Abrusan, G., Grundmann, N., Demester, L., and Makalowski, W. (2009). TEclass-a tool for automated classification of unknown eukaryotic transposable elements. *Bioinformatics* 25**,** 1329-1330.

Barrett, J.C., Fry, B., Maller, J., and Daly, M.J. (2005a). Haploview: analysis and visualization of LD and haplotype maps. *Bioinformatics* 21**,** 263-265.

Barrett, J.C., Fry, B., Maller, J., and Daly, M.J. (2005b). Haploview: analysis and visualization of LD and haplotype maps. *Bioinformatics* 21**,** 263-265.

Camacho, C., Coulouris, G., Avagyan, V., Ma, N., Papadopoulos, J., Bealer, K., and Madden, T.L. (2009). BLAST+: architecture and applications. *BMC Bioinformatics* 10**,** 421.

Chen, K.S., Li, F., Xu, C.J., Zhang, S.L., and Fu, C.X. (2004). An efficient macro-method of genomic DNA isolation from Actinidia chinensis leaves. *Yi Chuan* 26**,** 529-531.

Danecek, P., Auton, A., Abecasis, G., Albers, C.A., Banks, E., Depristo, M.A., Handsaker, R.E., Lunter, G., Marth, G.T., Sherry, S.T., Mcvean, G., Durbin, R., and Genomes Project Analysis, G. (2011). The variant call format and VCFtools. *Bioinformatics* 27**,** 2156-2158.

Edgar, R.C. (2004). MUSCLE: a multiple sequence alignment method with reduced time and space complexity. *BMC Bioinformatics* 5**,** 113.

Ellinghaus, D., Kurtz, S., and Willhoeft, U. (2008). LTRharvest, an efficient and flexible software for de novo detection of LTR retrotransposons. *BMC Bioinformatics* 9**,** 18.

Estill, J.C., and Bennetzen, J.L. (2009). The DAWGPAWS pipeline for the annotation of genes and transposable elements in plant genomes. *Plant Methods* 5**,** 8.

Gonzalez, J., Macpherson, J.M., and Petrov, D.A. (2009). A recent adaptive transposable element insertion near highly conserved developmental loci in *Drosophila melanogaster*. *Mol Biol Evol* 26**,** 1949-1961.

Hollister, J.D., Smith, L.M., Guo, Y.L., Ott, F., Weigel, D., and Gaut, B.S. (2011). Transposable elements and small RNAs contribute to gene expression divergence between *Arabidopsis thaliana* and *Arabidopsis lyrata*. *Proceedings of the National Academy of Sciences of the United States of America* 108**,** 2322-2327.

Kim, D., Paggi, J.M., Park, C., Bennett, C., and Salzberg, S.L. (2019). Graph-based genome alignment and genotyping with HISAT2 and HISAT-genotype. *Nature Biotechnology* 37**,** 1.

Kimura, M. (1980). A simple method for estimating evolutionary rates of base substitutions through comparative studies of nucleotide sequences. *Journal of Molecular Evolution* 16**,** 111-120.

Kofler, R., Betancourt, A.J., and Schlotterer, C. (2012). Sequencing of pooled DNA samples (Pool-Seq) uncovers complex dynamics of transposable element insertions in Drosophila melanogaster. *PLoS Genet* 8**,** e1002487.

Li, H., and Durbin, R. (2009). Fast and accurate short read alignment with Burrows-Wheeler transform. *Bioinformatics* 25**,** 1754-1760.

Li, H., and Durbin, R. (2010). Fast and accurate long-read alignment with Burrows-Wheeler transform. *Bioinformatics* 26**,** 589-595.

Nei, M. (1987). *Molecular evolutionary genetics.* PLENUM.

Niu, X.M., Xu, Y.C., Li, Z.W., Bian, Y.T., Hou, X.H., Chen, J.F., Zou, Y.P., Jiang, J., Wu, Q., Ge, S., Balasubramanian, S., and Guo, Y.L. (2019). Transposable elements drive rapid phenotypic variation in Capsella rubella. *Proc Natl Acad Sci U S A* 116**,** 6908-6913.

Ossowski, S., Schneeberger, K., Lucas-Lledo, J.I., Warthmann, N., Clark, R.M., Shaw, R.G., Weigel, D., and Lynch, M. (2010). The rate and molecular spectrum of spontaneous mutations in *Arabidopsis thaliana*. *Science* 327**,** 92-94.

Ou, S., and Jiang, N. (2018). LTR_retriever: A highly accurate and sensitive program for identification of long terminal repeat retrotransposons. *Plant Physiol* 176**,** 1410-1422.

Shen, J., Liu, J., Xie, K., Xing, F., Xiong, F., Xiao, J., Li, X., and Xiong, L. (2017). Translational repression by a miniature inverted-repeat transposable element in the 3′ untranslated region. *Nature Communications* 8**,** 14651.

Stuart, T., Eichten, S.R., Cahn, J., Karpievitch, Y.V., Borevitz, J.O., and Lister, R. (2016). Population scale mapping of transposable element diversity reveals links to gene regulation and epigenomic variation. *Elife* 5.

Tajima, F. (1989). Statistical method for testing the neutral mutation hypothesis by DNA polymorphism. *Genetics* 123**,** 585-595.

Trapnell, C., Roberts, A., Goff, L., Pertea, G., Kim, D., Kelley, D.R., Pimentel, H., Salzberg, S.L., Rinn, J.L., and Pachter, L. (2012). Differential gene and transcript expression analysis of RNA-seq experiments with TopHat and Cufflinks. *Nat Protoc* 7**,** 562-578.

Wang, L., He, L., Li, J., Zhao, J., Li, Z., and He, C. (2014). Regulatory change at Physalis Organ Size 1 correlates to natural variation in tomatillo reproductive organ size. *Nature communications* 5**,** 4271.

Wang, Y., Tang, H., Debarry, J.D., Tan, X., Li, J., Wang, X., Lee, T.H., Jin, H., Marler, B., Guo, H., Kissinger, J.C., and Paterson, A.H. (2012). MCScanX: a toolkit for detection and evolutionary analysis of gene synteny and collinearity. *Nucleic Acids Res* 40**,** e49.

Wicker, T., Sabot, F., Hua-Van, A., Bennetzen, J.L., Capy, P., Chalhoub, B., Flavell, A., Leroy, P., Morgante, M., Panaud, O., Paux, E., Sanmiguel, P., and Schulman, A.H. (2007). A unified classification system for eukaryotic transposable elements. *Nat Rev Genet* 8**,** 973-982.

Xu, X., Liu, X., Ge, S., Jensen, J.D., Hu, F., Li, X., Dong, Y., Gutenkunst, R.N., Fang, L., Huang, L., Li, J., He, W., Zhang, G., Zheng, X., Zhang, F., Li, Y., Yu, C., Kristiansen, K., Zhang, X., Wang, J., Wright, M., Mccouch, S., Nielsen, R., Wang, J., and Wang, W. (2011). Resequencing 50 accessions of cultivated and wild rice yields markers for identifying agronomically important genes. *Nat Biotechnol* 30**,** 105-111.

Xu, Z., and Wang, H. (2007). LTR_FINDER: an efficient tool for the prediction of full-length LTR retrotransposons. *Nucleic Acids Res* 35**,** W265-268.

Yang, Z.H. (2007). PAML 4: Phylogenetic analysis by maximum likelihood. *Molecular Biology and Evolution* 24**,** 1586-1591.

Yoo, S.D., Cho, Y.H., and Sheen, J. (2007). Arabidopsis mesophyll protoplasts: a versatile cell system for transient gene expression analysis. *Nat Protoc* 2**,** 1565-1572.
