# Supplementary material for: Transposable Elements: Distribution, Polymorphism, and Climate Adaptation in Populus
Source: Front Plant Sci. 2022 Feb 1;13:814718. doi: 10.3389/fpls.2022.814718 (PMC8843856; doi:10.3389/fpls.2022.814718)
Supplement: Supplementary file 11 [file Table_1.docx]

**Table S1.** Classification of transposable elements in the *Populus trichocarpa*, *P. tomentosa*, *P. alba × P. glandulosa* (84K), and *P. alba genome.* ^a)^

| Type | | *Populus trichocarpa* | | |  | | *Populus tomentosa* | | | |  | *Populus alba* × *Populus glandulosa* (84K) | | | |  | *Populus alba* | | | |
| --- | --- | --- | --- | --- | --- | --- | --- | --- | --- | --- | --- | --- | --- | --- | --- | --- | --- | --- | --- | --- |
|  |  | Number of elements | Length (bp) | % of genome |  | | Number of elements | Length (bp) | % of genome | |  | Number of elements | Length (bp) | % of genome | |  | Number of elements | Length (bp) | % of genome | |
| **Retroelemnets** | | 143,304 | 84,626,331 | 21.74% |  | | 144,272 | 87,384,773 | 22.00% | |  | 115,732 | 76,269,128 | 21.42% | |  | 145,681 | 102,375,790 | 24.55% | |
| LTR elements | Ty1/Copia | 36,731 | 19,332,460 | 20.22% | 4.97% |  | 39,112 | 21,180,137 | 20.32% | 5.33% |  | 31,333 | 18,742,343 | 19.94% | 5.26% |  | 35,291 | 21,999,696 | 22.51% | 5.28% |
|  | Gypsy/DIRS1 | 83,948 | 58,096,622 |  | 14.93% |  | 78,776 | 58,848,306 |  | 14.81% |  | 66,273 | 51,651,802 |  | 14.51% |  | 85,522 | 71,066,836 |  | 17.04% |
|  | others | 611 | 745,989 |  | 0.19% |  | 1,697 | 736,382 |  | 0.18% |  | 1,835 | 584,371 |  | 0.17% |  | 2,561 | 805,228 |  | 0.19% |
| SINEs | | 13,290 | 2,020,672 | 0.52% |  | | 19,807 | 2,994,500 | 0.75% | |  | 8,788 | 1,333,656 | 0.37% | |  | 13,247 | 2,348,541 | 0.56% | |
| LINEs | | 7,033 | 3,901,125 | 1.00% |  | | 5,040 | 3,503,090 | 0.88% | |  | 7,421 | 3,940,409 | 1.11% | |  | 8,716 | 5,865,074 | 1.41% | |
| **DNA transposons** | | 69,291 | 27,475,947 | 7.06% |  | | 57,334 | 24,189,384 | 6.09% | |  | 46,785 | 20,322,643 | 5.71% | |  | 63,399 | 24,960,956 | 5.99% | |
| **Rolling-circles** | | 72,595 | 25,541,484 | 6.56% |  | | 71,752 | 24,351,663 | 6.13% | |  | 60,348 | 19,730,040 | 5.54% | |  | 69,630 | 23,382,121 | 5.61% | |
| **Unclassified** | | 142,711 | 32,080,439 | 8.24% |  | | 125,016 | 30,845,645 | 7.76% | |  | 146,749 | 31,146,923 | 8.75% | |  | 135,339 | 37,236,122 | 8.93% | |
| **Total** | | - | 169,724,201 | 43.60% |  | | - | 166,771,465 | 41.98% | |  | - | 147,468,734 | 41.42% | |  | - | 187,954,989 | 45.08% | |
| **Satellites** | | 993 | 322,947 | 0.08% |  | | 431 | 63,941 | 0.02% | |  | 57 | 52,124 | 0.01% | |  | 212 | 290,659 | 0.07% | |
| **Simple repeats** | | 112,594 | 2,418,819 | 1.10% |  | | 129,010 | 4,861,440 | 1.22% | |  | 115,279 | 4,239,659 | 1.19% | |  | 134,368 | 5,313,072 | 1.27% | |
| **Low complexity** | | 20,890 | 1,008,291 | 0.26% |  | | 23,979 | 1,162,621 | 0.29% | |  | 21,394 | 1,025,151 | 0.29% | |  | 24,323 | 1,173,723 | 0.28% | |
| **Total** | | - | 173,474,258 | 45.04% |  | | - | 172,859,467 | 43.51% | |  | - | 152,785,668 | 42.91% | |  | - | 194,732,443 | 46.70% | |
| a) All repeat types were assigned according to homology to the Repbase database (version: 20170127; http://www.girinst.org/repbase). The names of the main classes of repetitive elements are shown in bold. The mitochondrion and chloroplast sequences were not counted. | | | | | | | | | | | | | | | | | | | |  |
