# Supplementary material for: Transposable Elements: Distribution, Polymorphism, and Climate Adaptation in Populus
Source: Front Plant Sci. 2022 Feb 1;13:814718. doi: 10.3389/fpls.2022.814718 (PMC8843856; doi:10.3389/fpls.2022.814718)
Supplement: Supplementary file 12 [file Table_2.docx]

| **Table S2.** The published re-sequencing genomes of *P. tomentosa* population used in this study. | | | |
| --- | --- | --- | --- |
|  |  |  |  |
| **Number** | **Accession name** | **Population** | **Accession ID** |
| 1 | 0009 | popNE | Raw sequence data of genome re-sequencing have been deposited in the Genome Sequence Archive in BIG Data Center, Beijing Institute of Genomics (BIG), Chinese Academy of Sciences, under accession number CRA000903 that is publicly accessible at http://bigd.big.ac.cn/gsa |
| 2 | 0010 | popNE |  |
| 3 | 0043 | popNE |  |
| 4 | 0057 | popNE |  |
| 5 | 0070 | popNE |  |
| 6 | 0074 | popNE |  |
| 7 | 0095 | popNE |  |
| 8 | 0096 | popNE |  |
| 9 | 1002 | popNE |  |
| 10 | 1016 | popNE |  |
| 11 | 1112 | popNE |  |
| 12 | 1201 | popNE |  |
| 13 | 1211 | popNE |  |
| 14 | 1223 | popNE |  |
| 15 | 1244 | popNE |  |
| 16 | 1278 | popNE |  |
| 17 | 1306 | popNE |  |
| 18 | 1328 | popNE |  |
| 19 | 1338 | popNE |  |
| 20 | 1354 | popNE |  |
| 21 | 1402 | popNE |  |
| 22 | 1407 | popNE |  |
| 23 | 1418 | popNE |  |
| 24 | 1501 | popNW |  |
| 25 | 1513 | popNW |  |
| 26 | 1702 | popNW |  |
| 27 | 1807 | popNW |  |
| 28 | 1808 | popNW |  |
| 29 | 2-19 | popNW |  |
| 30 | 2-3 | popNW |  |
| 31 | 2-5 | popNW |  |
| 32 | 2-8 | popNW |  |
| 33 | 3-10-1 | popNW |  |
| 34 | 3-16-28 | popNW |  |
| 35 | 3-24-3 | popNW |  |
| 36 | 3-25-1 | popNW |  |
| 37 | 3-4-2 | popNW |  |
| 38 | 3-5-3 | popNW |  |
| 39 | 3-50-2 | popNW |  |
| 40 | 3-57-3 | popNW |  |
| 41 | 3-7-1 | popNW |  |
| 42 | 3-9-3 | popNW |  |
| 43 | 3-92-1 | popNW |  |
| 44 | 3-98-1 | popNW |  |
| 45 | 3124 | popNW |  |
| 46 | 3210 | popNW |  |
| 47 | 3381 | popNW |  |
| 48 | 3425 | popNW |  |
| 49 | 3441 | popNW |  |
| 50 | 3601 | popNW |  |
| 51 | 3932 | popNW |  |
| 52 | 4101 | popNW |  |
| 53 | 4120 | popNW |  |
| 54 | 4129 | popNW |  |
| 55 | 4153 | popNW |  |
| 56 | 4201 | popNW |  |
| 57 | 4217 | popNW |  |
| 58 | 4221 | popNW |  |
| 59 | 4235 | popS |  |
| 60 | 4241 | popS |  |
| 61 | 4308 | popS |  |
| 62 | 4328 | popS |  |
| 63 | 4333 | popS |  |
| 64 | 4342 | popS |  |
| 65 | 4405 | popS |  |
| 66 | 4406 | popS |  |
| 67 | 4415 | popS |  |
| 68 | 4429 | popS |  |
| 69 | 5004 | popS |  |
| 70 | 5016 | popS |  |
| 71 | 5032 | popS |  |
| 72 | 5038 | popS |  |
| 73 | 5062 | popS |  |
| 74 | 5077 | popS |  |
| 75 | 5088 | popS |  |
| 76 | 5099 | popS |  |
| 77 | 5101 | popS |  |
| 78 | 5120 | popS |  |
| 79 | 5226 | popS |  |
| 80 | 5408 | popS |  |
| 81 | 6328 | popS |  |
| 82 | 6510 | popS |  |
| 83 | 8004 | popS |  |
| 84 | 8204 | popS |  |
| 85 | 8208 | popS |  |
| 86 | 8314 | popS |  |
| 87 | 9603 | popS |  |
