# Supplementary material for: Transposable Elements: Distribution, Polymorphism, and Climate Adaptation in Populus
Source: Front Plant Sci. 2022 Feb 1;13:814718. doi: 10.3389/fpls.2022.814718 (PMC8843856; doi:10.3389/fpls.2022.814718)
Supplement: Supplementary file 13 [file Table_3.docx]

| **Table S3.** The numbers of TE loci in the three populations. | |  |  |  |
| --- | --- | --- | --- | --- |
|  |  |  |  |  |
|  | popNE | popNW | popS | Three populations |
| TE loci with at least three reads per accession on average | 3,621 | 5,037 | 6,028 | 9,680 |
| Inserted TEs | 58,473 | 75,791 | 82,008 | 122,865 |
| Inserted TEs per accession (95% confidence intervals) | 549.10±2.07 | 508.48±2.03 | 714.41±2.05 | 333.05±1.99 |
| Population-specific TE loci | 231 | 685 | 1,401 | 263 |
