# Supplementary material for: Transposable Elements: Distribution, Polymorphism, and Climate Adaptation in Populus
Source: Front Plant Sci. 2022 Feb 1;13:814718. doi: 10.3389/fpls.2022.814718 (PMC8843856; doi:10.3389/fpls.2022.814718)
Supplement: Supplementary file 14 [file Table_4.docx]

| **Table S4.** Composition of polymorphic TEs in different populations. | | | |  |  |  |  |  |  |  |
| --- | --- | --- | --- | --- | --- | --- | --- | --- | --- | --- |
|  |  |  |  |  |  |  |  |  |  |  |
|  | **Percentage (number)** | | | | | | | | | **Sum** |
|  | **Helitron** | **hAT** | **Harbinger** | **other DNA** | **Copia** | **Gypsy** | **Ogre** | **other LTR** | **LINE** |  |
| All | 13.66% (1,322) | 4.83% (468) | 1.81% (175) | 25.72% (2,490) | 11.39% (1,103) | 11.85% (1,147) | 2.12% (205) | 28.12% (2,722) | 0.5% (48) | 9,680 |
| popNE | 11.82% (428) | 4.09% (148) | 2.35% (85) | 27.73% (1,004) | 11.24% (407) | 12.51% (453) | 1.44% (52) | 28.28% (1,024) | 0.55% (20) | 3,621 |
| popNW | 13.08% (659) | 5.02% (253) | 1.95% (98) | 26.05% (1,312) | 11.16% (562) | 11.08% (558) | 2.12% (107) | 29.18% (1,470) | 0.36% (18) | 5,037 |
| popS | 12.24% (738) | 5.19% (313) | 1.92% (116) | 23.87% (1,439) | 11.28% (680) | 11.75% (708) | 1.63% (98) | 31.52% (1,900) | 0.6% (36) | 6,028 |
| Shared | 11.47% (616) | 4.53% (243) | 2.09% (112) | 27.19% (1,460) | 9.46% (508) | 10.19% (547) | 1.45% (78) | 33.3% (1,788) | 0.34% (18) | 5,370 |
| popNE-specific | 15.58% (36) | 1.3% (3) | 1.30% (3) | 26.84% (62) | 15.58% (36) | 16.45% (38) | 0.87% (2) | 21.21% (49) | 0.87% (2) | 231 |
| popNW-specific | 20.73% (142) | 5.11% (35) | 2.63% (18) | 15.18% (104) | 18.39% (126) | 13.43% (92) | 7.3% (50) | 16.79% (115) | 0.44% (3) | 685 |
| popS-specific | 15.58% (222) | 6.07% (85) | 1.14% (16) | 18.34% (257) | 15.49% (217) | 13.13% (184) | 2.64% (37) | 26.55% (372) | 0.79% (11) | 1,401 |
