# Supplementary material for: Transposable Elements: Distribution, Polymorphism, and Climate Adaptation in Populus
Source: Front Plant Sci. 2022 Feb 1;13:814718. doi: 10.3389/fpls.2022.814718 (PMC8843856; doi:10.3389/fpls.2022.814718)
Supplement: Supplementary file 15 [file Table_5.docx]

| **Table S5.** Adaptive TE candidates. | |  |  |  |  |  |  |  |  |  |
| --- | --- | --- | --- | --- | --- | --- | --- | --- | --- | --- |
|  |  |  |  |  |  |  |  |  |  |  |
| **Number** | **Adaptive TE candidate in** | **TE ID** | **The predicted position of TE** | | |  | **TE insertion frequency in** | | |  |
|  |  |  | **chr** | **start** | **end** |  | **popNE (n=23)** | **popNW (n=35)** | **popS (n=29)** |  |
| 1 | popNE | Ogre-PT3_I-174967 | chr19 | 15420864 | 15420906 | 42 | 5 | 0 | 3 |  |
| 1 | popNW | Copia47-PTR_LTR-16896 | chr13 | 10264842 | 10264944 | 102 | 1 | 11 | 3 |  |
| 2 | popNW | Helitron-N3_PTr-68896 | chr19 | 16106211 | 16106273 | 62 | 3 | 1 | 0 |  |
| 3 | popNW | Ogre-PT2_LTR-59150 | chr15 | 6228042 | 6228282 | 240 | 0 | 21 | 1 |  |
| 4 | popNW | PTr-3-152088 | chr16 | 10387055 | 10387128 | 73 | 0 | 5 | 11 |  |
| 5 | popNW | Helitron-N1_PTr-116603 | chr19 | 9279864 | 9279934 | 70 | 5 | 4 | 3 |  |
| 6 | popNW | PTr-3-156683 | chr18 | 12729934 | 12730009 | 75 | 1 | 1 | 1 |  |
| 7 | popNW | DNA-3-1_PTr-97043 | chr8 | 1227587 | 1227678 | 91 | 1 | 1 | 0 |  |
| 8 | popNW | PTr-3-96831 | chr10 | 8116718 | 8116826 | 108 | 0 | 13 | 1 |  |
| 9 | popNW | PTr-3-146618 | chr10 | 8116726 | 8116826 | 100 | 10 | 15 | 3 |  |
| 10 | popNW | Ogre-PT3_LTR-20005 | chr18 | 11117543 | 11117612 | 69 | 2 | 6 | 1 |  |
| 11 | popNW | Helitron-N3_PTr-107488 | chr18 | 12705388 | 12705469 | 81 | 0 | 25 | 2 |  |
| 12 | popNW | PTr-3-147886 | chr4 | 4917668 | 4917765 | 97 | 2 | 7 | 6 |  |
| 13 | popNW | DNA-3-1_PTr-34583 | chr18 | 7130300 | 7130400 | 100 | 1 | 2 | 1 |  |
| 14 | popNW | PTr-3-44781 | chr1 | 15606075 | 15606143 | 68 | 0 | 9 | 4 |  |
| 15 | popNW | EnSpm1B_PT-123912 | chr13 | 1974598 | 1974679 | 81 | 0 | 1 | 0 |  |
| 16 | popNW | DNA-3-1_PTr-96553 | chr8 | 1227572 | 1227678 | 106 | 3 | 23 | 0 |  |
| 17 | popNW | PTr-3-110384 | chr16 | 7389882 | 7389913 | 31 | 6 | 11 | 1 |  |
| 18 | popNW | ENSPM1_PT-119077 | chr5 | 22630261 | 22630318 | 57 | 0 | 7 | 1 |  |
| 19 | popNW | hAT-5_PTr-56704 | chr7 | 15769326 | 15769395 | 69 | 0 | 10 | 0 |  |
| 20 | popNW | PTr-3-31707 | chr19 | 1652682 | 1652795 | 113 | 1 | 3 | 2 |  |
| 21 | popNW | hAT-1N_PTr-36757 | chr19 | 12016757 | 12016813 | 56 | 0 | 12 | 1 |  |
| 22 | popNW | PTr-3-21506 | chr7 | 3328075 | 3328103 | 28 | 9 | 19 | 15 |  |
| 23 | popNW | PTr-3-57557 | chr1 | 15606073 | 15606159 | 86 | 13 | 23 | 17 |  |
| 24 (TE2) | popNW | Helitron-N4_PTr-50444 | chr5 | 17018780 | 17018810 | 30 | 0 | 1 | 0 |  |
| 1 | popS | Gypsy-79_PTr-LTR-35996 | chr15 | 6412935 | 6412975 | 40 | 3 | 3 | 5 |  |
| 2 | popS | Copia-54_PTr-LTR-89983 | chr4 | 8817090 | 8817231 | 141 | 7 | 18 | 7 |  |
| 3 | popS | Gypsy-73_PTr-LTR-166661 | chr16 | 8440963 | 8441078 | 115 | 4 | 6 | 6 |  |
| 4 | popS | Harbinger1_PTr-158507 | chr18 | 2547130 | 2547348 | 218 | 3 | 8 | 3 |  |
| 5 | popS | DNA-3-1_PTr-95528 | chr1 | 5447090 | 5447146 | 56 | 1 | 1 | 22 |  |
| 6 | popS | PTr-3-56987 | chr2 | 21890687 | 21890733 | 46 | 1 | 1 | 2 |  |
| 7 | popS | Helitron-N3_PTr-128304 | chr19 | 1545820 | 1545930 | 110 | 1 | 1 | 1 |  |
| 8 | popS | DNA-3-1_PTr-92673 | chr13 | 4365457 | 4365505 | 48 | 2 | 1 | 1 |  |
| 9 | popS | DNA-3-2_PTr-125386 | chr6 | 19182185 | 19182262 | 77 | 2 | 4 | 2 |  |
| 10 | popS | Copia-56_PTr-LTR-56261 | chr2 | 19540519 | 19540614 | 95 | 6 | 6 | 9 |  |
| 11 | popS | PTr-3-90669 | chr5 | 7001853 | 7001935 | 82 | 2 | 1 | 4 |  |
| 12 | popS | DNA-3-1_PTr-156840 | chr2 | 19847535 | 19847633 | 98 | 5 | 6 | 4 |  |
| 13 | popS | Gypsy-28_PTr-LTR-75082 | chr2 | 21853140 | 21853245 | 105 | 1 | 1 | 19 |  |
| 14 | popS | PTr-3-51824 | chr16 | 13561210 | 13561255 | 45 | 0 | 0 | 4 |  |
| 15 | popS | Helitron-N2_PTr-156743 | chr7 | 7091607 | 7091673 | 66 | 0 | 1 | 1 |  |
| 16 | popS | Gypsy-79_PTr-LTR-18261 | chr19 | 11319423 | 11319502 | 79 | 1 | 3 | 1 |  |
| 17 | popS | Copia-3_PTri-I-108777 | chr16 | 2308504 | 2308534 | 30 | 1 | 1 | 1 |  |
| 18 | popS | DNA-3-1_PTr-21025 | chr1 | 35361701 | 35361796 | 95 | 0 | 6 | 4 |  |
| 19 | popS | ENSPM1_PT-28431 | chr1 | 38911007 | 38911214 | 207 | 3 | 0 | 16 |  |
| 20 | popS | DNA-3-3_PTr-125150 | chr8 | 9496245 | 9496344 | 99 | 2 | 1 | 14 |  |
| 21 | popS | DNA-3-1_PTr-123875 | chr4 | 15094058 | 15094088 | 30 | 4 | 1 | 8 |  |
| 22 | popS | PTr-3-64591 | chr10 | 17772754 | 17772831 | 77 | 17 | 0 | 14 |  |
| 23 | popS | Gypsy-73_PTr-LTR-43088 | chr16 | 6824582 | 6824662 | 80 | 10 | 24 | 12 |  |
| 24 | popS | PTr-3-94961 | chr16 | 1853984 | 1854071 | 87 | 2 | 3 | 1 |  |
| 25 | popS | Gypsy-78_PTr-LTR-34139 | chr12 | 1509162 | 1509285 | 123 | 0 | 1 | 1 |  |
| 26 | popS | DNA-3-1_PTr-62210 | chr2 | 19848087 | 19848159 | 72 | 0 | 3 | 1 |  |
| 27 | popS | DNA-3-1_PTr-97140 | chr19 | 13171494 | 13171585 | 91 | 1 | 2 | 1 |  |
| 28 | popS | DNA-3-1_PTr-38248 | chr1 | 42095689 | 42095898 | 209 | 0 | 0 | 7 |  |
| 29 | popS | Gypsy-73_PTr-LTR-103796 | chr19 | 9407015 | 9407086 | 71 | 1 | 1 | 1 |  |
| 30 (TE1) | popS | Helitron-N3_PTr-21548 | chr6 | 22887823 | 22888661 | 837 | 0 | 0 | 2 |  |
| 31 | popS | Copia-3_PTri-I-144419 | chr11 | 5791307 | 5791423 | 116 | 0 | 0 | 1 |  |
| 32 | popS | POPGY1_I-15138 | chr18 | 2709623 | 2709656 | 33 | 0 | 0 | 1 |  |
| 33 | popS | Helitron-N1_PTr-151860 | chr1 | 27282028 | 27282076 | 48 | 2 | 1 | 1 |  |
| 34 | popS | Copia-3_PTri-I-42222 | chr6 | 11950504 | 11950534 | 30 | 2 | 14 | 9 |  |
| 35 | popS | PTr-3-132462 | chr7 | 14047986 | 14048078 | 92 | 1 | 0 | 1 |  |
| 36 | popS | Helitron-N1_PTr-131230 | chr1 | 15161900 | 15161992 | 92 | 0 | 0 | 1 |  |
| 37 | popS | hAT-5_PTr-56704 | chr7 | 15769326 | 15769395 | 69 | 0 | 10 | 21 |  |
| 38 | popS | Gypsy-78_PTr-LTR-167630 | chr12 | 1505788 | 1505863 | 75 | 0 | 1 | 2 |  |
| 39 | popS | PTr-3-21506 | chr7 | 3328075 | 3328103 | 28 | 9 | 19 | 15 |  |
| 40 | popS | PTr-3-26900 | chr4 | 16463341 | 16463394 | 53 | 1 | 2 | 7 |  |
| 41 | popS | EnSpm1B_PT-59829 | chr12 | 905734 | 905781 | 47 | 0 | 0 | 1 |  |
| 42 | popS | Gypsy-79_PTr-LTR-3214 | chr6 | 19447733 | 19447850 | 117 | 0 | 0 | 8 |  |
| 43 | popS | DNA-3-1_PTr-113234 | chr1 | 42080836 | 42081057 | 221 | 0 | 2 | 1 |  |
| 44 | popS | PTr-3-157566 | chr17 | 12377238 | 12377333 | 95 | 0 | 0 | 1 |  |
| 45 | popS | Helitron-N3_PTr-158729 | chr12 | 10332826 | 10332879 | 53 | 0 | 2 | 1 |  |
|  |  |  |  |  |  |  |  |  |  |  |
|  |  |  |  |  |  |  |  |  |  |  |
|  |  |  |  |  |  |  |  |  |  |  |
|  |  |  |  |  |  |  |  |  |  |  |
|  |  |  |  |  |  |  |  |  |  |  |
|  |  |  |  |  |  |  |  |  |  |  |
|  |  |  |  |  |  |  |  |  |  |  |
