# Supplementary material for: Transposable Elements: Distribution, Polymorphism, and Climate Adaptation in Populus
Source: Front Plant Sci. 2022 Feb 1;13:814718. doi: 10.3389/fpls.2022.814718 (PMC8843856; doi:10.3389/fpls.2022.814718)
Supplement: Supplementary file 16 [file Table_6.docx]

| **Table S6.** Validation of TEs in the populations. | | | | | | | | | | | | | | | | |
| --- | --- | --- | --- | --- | --- | --- | --- | --- | --- | --- | --- | --- | --- | --- | --- | --- |
| **ID** | **Chromosome** | **Position on the left** | **Position on the right** | **Length of TE (bp)** | **popNE accessions** | | | | **popNW accessions** | | | | **popS accessions** | | | |
|  |  |  |  |  | **mby-0057** | **mby-0096** | **mby-1306** | **mby-1354** | **mby-2-3** | **mby-3-50-2** | **mby-3425** | **mby-4217** | **mby-4235** | **mby-4405** | **mby-6328** | **mby-9603** |
| Gypsy-28_PTr-LTR-75082 | chr2 | 21853140 | 21853245 | 105 | 0 | 0 | 0 | 0 | 1 | 1 | 1 | 0 | 0 | 0 | 0 | 0 |
| Copia-54_PTr-LTR-89983 | chr4 | 8817090 | 8817231 | 141 | 0 | 0 | 0 | 1 | 0 | 0 | 0 | 0 | 0 | 0 | 0 | 1 |
| Helitron-N4_PTr-50444 | chr5 | 17018780 | 17018810 | 30 | 1 | 1 | 0 | 1 | 0 | 0 | 0 | 0 | 1 | 1 | 0 | 1 |
| Helitron-N3_PTr-21548 | chr6 | 22887823 | 22888661 | 837 | 1 | 1 | 1 | 1 | 0 | 0 | 0 | 0 | 1 | 1 | 1 | 1 |
| Gypsy-79_PTr-LTR-3214 | chr6 | 19447733 | 19447850 | 117 | 1 | 1 | 0 | 1 | 1 | 1 | 0 | 1 | 1 | 1 | 0 | 1 |
| hAT-5_PTr-56704 | chr7 | 5769326 | 5769395 | 69 | 0 | 0 | 0 | 0 | 1 | 1 | 0 | 1 | 0 | 0 | 0 | 0 |
| DNA-3-1_PTr-97043 | chr8 | 1227587 | 1227678 | 91 | 1 | 1 | 1 | 1 | 1 | 1 | 1 | 1 | 1 | 1 | 1 | 1 |
| DNA-3-1_PTr-96553 | chr8 | 1227572 | 1227678 | 106 | 1 | 1 | 1 | 1 | 0 | 0 | 0 | 0 | 1 | 1 | 1 | 1 |
| EnSpm1B_PT-123912 | chr13 | 1974598 | 1974679 | 81 | 1 | 1 | 0 | 1 | 0 | 0 | 0 | 0 | 1 | 1 | 0 | 1 |
| Gypsy-79_PTr-LTR-35996 | chr15 | 6412935 | 6412975 | 40 | 0 | 0 | 0 | 1 | 1 | 1 | 1 | 1 | 0 | 0 | 0 | 1 |
| Gypsy-73_PTr-LTR-166661 | chr16 | 8440963 | 8441078 | 115 | 0 | 0 | 0 | 0 | 1 | 1 | 1 | 1 | 0 | 0 | 0 | 0 |
| Harbinger1_PTr-158507 | chr18 | 2547130 | 2547348 | 218 | 1 | 0 | 1 | 1 | 1 | 1 | 0 | 0 | 1 | 0 | 1 | 1 |
| Ogre-PT3_I-174967 | chr19 | 15420864 | 15420906 | 42 | 0 | 0 | 1 | 0 | 0 | 0 | 0 | 0 | 0 | 0 | 1 | 0 |
| PTr-3-31707 | chr19 | 1652682 | 1652795 | 113 | 0 | 0 | 1 | 1 | 0 | 0 | 1 | 0 | 0 | 0 | 1 | 1 |
| Green indicates correct validation, yellow indicates TE length is slightly different than the real length and red indicates an incorrectly predicted TE. 1 or 0 indicates that there is and is not a TE, respectively. | | | | | | | | | | | | | | | | |
