# Supplementary material for: Transposable Elements: Distribution, Polymorphism, and Climate Adaptation in Populus
Source: Front Plant Sci. 2022 Feb 1;13:814718. doi: 10.3389/fpls.2022.814718 (PMC8843856; doi:10.3389/fpls.2022.814718)
Supplement: Supplementary file 17 [file Table_7.docx]

| **Table S7.** Primers used in this study. | |
| --- | --- |
| **Primer name** | **Primer sequence (5'–3')** |
| **Gypsy-28_PTr-LTR-75082 F** | **CAAGGCTTGTTTGGGCTTAA** |
| **Gypsy-28_PTr-LTR-75082 R** | **TACAAAAACTGACTCAAAACCT** |
| **Copia-54_PTr-LTR-89983 F** | **GGACACTCAACAATTCATCC** |
| **Copia-54_PTr-LTR-89983 R** | **GTCCTCTACCTCAGTCGAA** |
| **Helitron-N4_PTr-50444 F** | **TAATAACCCTAAAGAAAGC** |
| **Helitron-N4_PTr-50444 R** | **TTTTTTTTTTTTGCTTTCTTTAG** |
| **Helitron-N3_PTr-21548 F** | **TTTTTTTTATCTTTAATTTTTTTTCA** |
| **Helitron-N3_PTr-21548 R** | **AATATGGTTTCTAGCTTTCACTAG** |
| **Gypsy-79_PTr-LTR-3214 F** | **ACTGTTAAATTTTATAATCTTG** |
| **Gypsy-79_PTr-LTR-3214 R** | **AAAATATTGGAAGAAAATA** |
| **hAT-5_PTr-56704 F** | **TGTTACATAGTTTGAGGAG** |
| **hAT-5_PTr-56704 R** | **TTGCAAATACTGATACGTA** |
| **DNA-3-1_PTr-97043 F** | **AAAAGTGTTTTTCAATTTATTTT** |
| **DNA-3-1_PTr-97043 R** | **AAAAGTAGTTTTTTTGAAAAAT** |
| **DNA-3-1_PTr-96553 F** | **CATAACCAAATATTAAAAAGTGTTT** |
| **DNA-3-1_PTr-96553 R** | **AAAAGTAGTTTTTTTGAAAAATAAATT** |
| **EnSpm1B_PT-123912 F** | **AAATTTAAAAAAAGTAAAAAAATGATGA** |
| **EnSpm1B_PT-123912 R** | **ATTTTACCAACAGAATTTATTCC** |
| **Gypsy-79_PTr-LTR-35996 F** | **GTTAGTTTGTCTAAAATTT** |
| **Gypsy-79_PTr-LTR-35996 R** | **TTTACCAAGAAAAACTTT** |
| **Gypsy-73_PTr-LTR-166661 F** | **GGTTCCTAGTGACCAGAATA** |
| **Gypsy-73_PTr-LTR-166661 R** | **AAGAAAAATGTATAATGTCTATTTGG** |
| **Harbinger1_PTr-158507 F** | **CAAAGGTGTGAGAGAGTAATG** |
| **Harbinger1_PTr-158507 R** | **AGATGAGTATTTCCTTGGTCG** |
| **Ogre-PT3_I-174967 F** | **TCGCCACTAGAATTGCC** |
| **Ogre-PT3_I-174967 R** | **ACATTTGCTTACTAGTATTGCA** |
| **PTr-3-31707 F** | **TATTTTGACTTTTAATTTTTTTTTTTTAA** |
| **PTr-3-31707 R** | **AAATTAGAAATCACTCAATCAATTG** |
| **WRKY18 fl F BamHI** | **agctcggtacccgggGACCAACTTCAGACCATAGTGC** |
| **WRKY18 fl R XbaI** | **gcctgcaggtcgact TAAGAAAAGAATTAATTAAACTCT** |
| **WRKY18 cds F KpnI** | **attcgagctcggtacATGTCAATGAAACTCAAGCTCAAGA** |
| **WRKY18 cds R BamHI** | **aggtcgactctagagTCACAATTTCTCAATCCGAGTCTG** |
| **WRKY18 del Helitron fl inside F** | **tgatgatcagctatcTTGATGATTCTTCACAGGCTGGT** |
| **WRKY18 del Helitron fl inside R** | **gtgaagaatcatcaaGATAGCTGATCATCACCTTTCACA** |
| **WRKY18-3'UTR fl F** | **ACACTAGTACTGTTGTAGAGA** |
| **WRKY18-3'UTR fl R** | **GTAACTCAATGCTCTCCAAAT** |
| **WRKY18-3'UTR del-1 F** | **agctcggtacccgggGAATTTAGAGTTTTATAGACACTAT** |
| **WRKY18-3'UTR del-1 R** | **gcctgcaggtcgactTAAGAAAAGAATTAATTAAACTCTT** |
| **WRKY18-3'UTR del-2 F** | **agctcggtacccgggGGTTGAAAACACTAGTACTGTTGTA** |
| **WRKY18-3'UTR del-2 R** | **gcctgcaggtcgactATTTAATTTTTTTTTCAAACTTATA** |
| **Helitron F** | **TTTTTTTTATCTTTAATTTTTTTTCA** |
| **Helitron R** | **AATATGGTTTCTAGCTTTCACTAG** |
| **Mut-1 F** | **AACTAGGTTGAACATAACTTCT** |
| **Mut-1 R** | **TTATGAAGTATAGTGTCCTCA** |
| **Mut-2 F** | **GGTGAATGAATAATTTTTCATATGCT** |
| **Mut-2 R** | **ATGTAAACAACAACCCTCTTCAGT** |
